# Differential endothelial cell gene expression by African Americans *versus *Caucasian Americans: a possible contribution to health disparity in vascular disease and cancer

**DOI:** 10.1186/1741-7015-9-2

**Published:** 2011-01-11

**Authors:** P Wei, LC Milbauer, J Enenstein, J Nguyen, W Pan, RP Hebbel

**Affiliations:** 1 Vascular Biology Center, Department of Medicine, Medical School, University of Minnesota, Minneapolis, MN 55455, USA; 2 Division of Biostatistics, School of Public Health, University of Minnesota, Minneapolis, MN 55455, USA; 3 Division of Hematology-Oncology-Transplantation, Department of Medicine, Medical School, University of Minnesota, Minneapolis, MN 55455, USA; 4 Division of Biostatistics and Human Genetics Center, School of Public Health, University of Texas Health and Science Center at Houston, Houston, TX 77030, USA

## Abstract

**Background:**

Health disparities and the high prevalence of cardiovascular disease continue to be perplexing worldwide health challenges. This study addresses the possibility that genetic differences affecting the biology of the vascular endothelium could be a factor contributing to the increased burden of cardiovascular disease and cancer among African Americans (AA) compared to Caucasian Americans (CA).

**Methods:**

From self-identified, healthy, 20 to 29-year-old AA (n = 21) and CA (n = 17), we established cultures of blood outgrowth endothelial cells (BOEC) and applied microarray profiling. BOEC have never been exposed to *in vivo *influences, and their gene expression reflects culture conditions (meticulously controlled) and donor genetics. Significance Analysis of Microarray identified differential expression of single genes. Gene Set Enrichment Analysis examined expression of pre-determined gene sets that survey nine biological systems relevant to endothelial biology.

**Results:**

At the highly stringent threshold of False Discovery Rate (FDR) = 0, 31 single genes were differentially expressed in AA. *PSPH *exhibited the greatest fold-change (AA > CA), but this was entirely accounted for by a homolog (*PSPHL*) hidden within the *PSPH *probe set. Among other significantly different genes were: for AA > CA, *SOS1, AMFR, FGFR3; and for AA < CA, ARVCF, BIN3, EIF4B. *Many more (221 transcripts for 204 genes) were differentially expressed at the less stringent threshold of FDR <.05. Using the biological systems approach, we identified shear response biology as being significantly different for AA versus CA, showing an apparent tonic increase of expression (AA > CA) for 46/157 genes within that system.

**Conclusions:**

Many of the genes implicated here have substantial roles in endothelial biology. Shear stress response, a critical regulator of endothelial function and vascular homeostasis, may be different between AA and CA. These results potentially have direct implications for the role of endothelial cells in vascular disease (hypertension, stroke) and cancer (via angiogenesis). Also, they are consistent with our over-arching hypothesis that genetic influences stemming from ancestral continent-of-origin could impact upon endothelial cell biology and thereby contribute to disparity of vascular-related disease burden among AA. The method used here could be productively employed to bridge the gap between information from structural genomics (for example, disease association) and cell function and pathophysiology.

## Background

Despite the enormous advances over the last century in the understanding of, and the ability to therapeutically manipulate, medical biology, both health disparities and the high prevalence of cardiovascular (including cerebrovascular) disease continue to be perplexing, worldwide medical challenges. From a world health perspective, [1] health disparities are evident comparing continents, countries, regions, and population subgroups defined, for example, by socioeconomic factors or ethnic/racial group. The reasons these exist are legion, but they basically fall into the categories of environment (in the broadest sense) and genetics. So understanding the basis for extant health disparities is (or will be) a goal of health delivery efforts worldwide.

The present study addresses a specific case of health disparity that is particularly amenable to analysis, the higher burden of cardiovascular disease borne by those of African ancestry who reside within the United States. In so doing, we illustrate the feasibility of a novel investigational approach that offers a way to bridge the current gap between the information provided by structural genomics (for example, identification of loci, genes, alleles, haplotypes associated with disease or disease risk) and the actual consequent impact upon cellular biology and disease pathophysiology. Thus, by demonstrating a way to link these two distinct facets of modern medial biology for vascular disease, the present approach may be very useful. For example, it could help tease out the enormously confounding effect of inter-individual epigenetic changes on attempts to associate a locus with a disease phenotype.

### Health disparity

Worldwide, coronary and cerebrovascular disease account for approximately 20% of deaths, an estimated 7.2 and 5.7 million people annually, and they are the two most common causes of death in high- and middle-income countries [1]. This proportion rises to approximately 30% if all cardiovascular disease types (for example, hypertension) are included. Even in low-income countries, cardiovascular disease is exceeded as the cause of death only by infectious diseases (in particular malaria, diarrheal diseases, tuberculosis and HIV) [1].

Within the United States there are significant health disparities between African Americans (AA) and Caucasian Americans (CA). Notably, AA have a 2.4-fold higher incidence of stroke [2] and an approximately 50% increase in prevalence of hypertension, the latter affecting approximately 31% of AA [3,4]. This same disparity exists in the United Kingdom [5], and a local study verifies that it occurs in our own region from which the present study subjects were drawn [6]. In addition, AA display an increased prevalence of cardiovascular co-morbidities that contribute to pathogenesis in the general population [7]. For example, obesity has a 50% higher prevalence and affects approximately 45% of AA [8]. Correspondingly, AA have a two- to three-fold higher prevalence of type-2 diabetes so that it affects approximately 12% of AA [9], and they have an increased incidence of smoking, physical inactivity, and peripheral artery disease [10]. In addition to bearing the burden of a higher prevalence of cardiovascular and cerebrovascular disease, AA tend to develop such clinical diseases at a younger age than do CA (see Discussion). Disparities in cancer are addressed in the Discussion.

The debate as to what relative degree environment versus genetics causes these disparities is ongoing and vigorous.

### Environmental influences

The many factors interposed between racial identification (see Discussion) and any health issue provide a perplexing spectrum of possible non-genetic routes to disease disparity [11]. Examples include nutrition and exposures, access to health care and disparities in its delivery, social conditions and lifestyle choices, and so on. For cardiovascular health, it is telling that urbanization and its accompanying adoption of Western lifestyles are implicated in the accelerating development of hypertension and its comorbidities within Africa [12]. Likewise, the African diaspora established parallel gradients of hypertension and comorbid risk factors from West Africa to the Caribbean to North America [13,14]. Even within the United States itself there are notable regional differences in stroke mortality rate among AA [15]. Thus, the tremendous influence of environmental factors is indisputable.

### Genetic influences

The present study, however, was conducted considering the possibility that the exaggerated burden among AA of cardiovascular disease, and perhaps even that of cancer, could stem in part from genetic determinants. Indeed, historical genetic studies indicated that ancestral continent-of-origin can be genetically identified [16]. Application of modern methods (for example, identification of single nucleotide polymorphisms [SNPs], haplotypes, copy-number variable loci, genetic diversity, frequency of non-beneficial SNPs) confirm an East African origin for modern humans with the spread of the human genome to the rest of the world [17-19]. In turn, modern populations can carry genes that confer altered risk (higher or lower) for disease burden [20]. Indeed, newer methods such as discovery of disease association through admixture mapping have implicated specific loci influencing, for example, hypertension [21,22], blood lipid levels [23], obesity and type-2 diabetes [24]. Such studies of AA versus CA have been extremely helpful in identifying the relationship between such loci and continent-of-origin.

### Gene-environment interactions

Environmental and genetic influences do not occur independently; rather, gene-environment interactions contribute to disease variation [25]. Dramatic examples of this in Africa include the sickle mutation, the Duffy mutation, and *APOL1 *variants conferring protection against *P. falciparum*, and *P. vivax*, and *Trypanosoma *disease, respectively. On the other hand, gene variants that are hypothesized to have been of benefit in ancestral Africa can exert a harmful effect in modern societies. For example, a *CYP3A5 *allele which may have been advantageous in hot ancestral regions is now associated with salt-sensitivity hypertension in modern AA [26]. Another example is, in principle, found in the "thrifty gene" theory which posits that maximized calorie storage was advantageous ancestrally, but under modern conditions of more abundant food availability, obesity and type-2 diabetes (insulin resistance) are epidemic problems [9].

Thus, obesity is substantially influenced by genetics and is now understood to be a polygenic, complex disease with very significant gene-environment interaction [27]. Likewise, the relevance of gene-environment interactions has been emphasized for hypertension [28], stroke [29], cardiovascular disease [30], as well as metabolic syndrome, diabetes and atherosclerosis [31]. Furthermore, sequence-independent examples of this have been proposed whereby environmental influences exerted during development, for example, by poor prenatal nutrition, could establish durable, even trans-generational, effects on disease risk among AA through epigenetic mechanisms [32]. At the least, it seems likely that such effects exerted in childhood can cause a child to embark on the road to eventual cardiovascular disease [28].

### The endothelial cell

The vascular endothelial cell is a universal participant in vascular diseases, and for that matter in many "non-vascular" diseases, for example, immune and infectious inflammatory diseases. Therefore, the endothelial cell could be an executor in fulfilment of any potential influence of genomic effects relevant to health discrepancies. Indeed, the endothelial cell is unique in being a critical participant in, and regulator of, multiple vascular functions, as well as comprising the major biological linkage between them. Examples include inflammation biology, governance of the pro- versus anti-coagulant balance, and regulation of vascular tone, among others. These processes and their proximate regulatory mechanisms interact in complex ways, so the functional/physiologic impact of even a precisely known allelic association is not necessarily accurately predictable. Hence, verification of genomic implications on cell biology is of vital importance for understanding pathophysiology, and such information can reveal therapeutic options and even inform pharmaceutical development.

Regarding vascular disease, AA exhibit a variety of findings consistent with endothelial dysfunction, in particular abnormal nitric oxide(NO)-dependent vasorelaxation [33,34]. Consistent with this, ethnicity affects prevalence of clinically relevant variants of endothelial nitric oxide synthase (eNOS) [35]. Also, cultured umbilical cord endothelial cells from AA babies revealed hints of eNOS malfunction (uncoupling) [36].

### Rationale

The present study reflects our overarching interest in the concept that genetically-influenced, inter-individual differences in endothelial cell biology contribute to the heterogeneity of clinical phenotype evident in vascular diseases. As a general concept relating genomics and health, this is an accepted and compelling model for complex diseases. Regarding endothelial biology specifically, we previously employed the present approach to reveal gene expression differences coincident with a corresponding exaggerated endothelial cell response to inflammation signaling among the subgroup of children with sickle cell anemia who develop arterial occlusive disease in the Circle of Willis at the base of the brain [37]. Similarly, the present study was enabled by the technology we previously devised [37] that allows production of robust cultures of reporter endothelial cells (BOEC, blood outgrowth endothelial cells) from peripheral blood obtained from specific, phenotypically-defined individuals, in the present case, those self-identified by race as being AA or CA. We chose this device for subject group assignment because it was the method used for seminal epidemiologic studies of stroke and hypertension prevalence among AA [2,3], and we wished the present results to be directly relevant to such studies. Caveats regarding this approach for subject group assignment, as well as the very concept of race, are presented in the Discussion.

## Methods

All aspects of this study were performed with the approval of and monitoring by the Institutional Review Board at the University of Minnesota and were in compliance with the Helsinki Declaration.

### Subjects

Eligible volunteer subjects were between 20 and 29 years of age, inclusive, because we wanted our study to focus on a young, healthy population. All subjects claimed to be healthy, to not have any known cardiovascular disease, and to not be taking heart or lipid-lowering or blood pressure medications. We chose subjects who self-identified as being CA or AA, and they were included only if they stated that both parents were also CA or AA, respectively. In Minnesota, CA are overwhelmingly of European origin, largely from central and northern Europe. We excluded potential subjects who were from coastal East Africa for two reasons. First, the significant influx of East Africans to the upper Midwest has been a recent phenomenon, largely occurring after the seminal studies of AA cardiovascular health disparity, including the one done locally. Second, the gene pool in East Africa differs somewhat from that of Central/West, sub-Saharan Africa (the ancestral origin of most African Americans), as evidenced by the former's low frequency of Duffy negative status and virtual absence of the sickle mutation [17-19].

Of the 45 subjects providing blood samples for this study, we subsequently excluded three AA and four CA because their BOEC cultures failed quality control testing (see below). The remaining 38 subjects (12 AA males, 9 AA females, 8 CA males, and 9 CA females) were grouped to create two subject groups, AA (n = 21) and CA (n = 17). The AA group included two individuals with sickle trait (tested for on each subject), the expected prevalence, but none with sickle cell anemia.

### Endothelial culture (BOEC, blood outgrowth endothelial cells)

Subjects donated 50 to 100 ml of citrated peripheral blood that we immediately used to obtain peripheral blood mononuclear cells that were then placed in a special culture system (on collagen I; with multiple endothelial growth factors) [38]. The outgrowth BOEC from this long-term culture are mature, fully differentiated endothelial cells having unambiguous identity (by phenotypic, functional, gene expression, and electron microscopic criteria); and their population uniformity (100% BOEC) has been established [38-41]. Although BOEC cultures ultimately reach approximately 10^18^-fold expansion [38], for the present study cells were expanded only to approximately 3 × 10^7 ^cells (representing approximately 10^6^-fold overall expansion). BOEC were harvested four hours after the last change of culture medium and when they were 85 to 90% confluent. Each culture was meticulously performed by the same, highly-trained individual, ensuring that each step was always executed in the same manner. Culture medium components were obtained prior to project onset so that the same lot of reagents could be used for all cultures in this study. Aliquots of BOEC were taken for experimental use, quality control tests, and for cryopreservation.

Our quality control protocol is to verify that BOEC cultures are morphologically endothelial (cobblestone), that the cells are positive for three endothelial markers (CD31, VE-cadherin, P1H12), and that the cells are negative for monocyte/myeloid markers (CD14, CD45) as well as a probable marker of endothelial progenitor cells (CD133). We obtain cytogenetics analysis on each culture to ensure the BOEC are not characterized by culture-acquired clonal chromosomal abnormalities. Failure to meet the latter criterion is the reason seven subjects were excluded from data analysis.

Importantly, BOEC are not the so-called "EPC" that have been repeatedly (and largely erroneously) described in the literature. Rather, they are the progeny of a circulating, marrow-derived, transplantable, true endothelial progenitor cell [38]. Thus, BOEC themselves have never been influenced by *in vivo *modifiers such as blood milieu or tissue-specific signaling. Unlike other endothelial types, BOEC retain their phenotype at high degrees of expansion. Since they cryopreserve very well, aliquots from these cultures were saved at time of harvest for potential, future follow-up studies. Our previous validation studies of the present methods demonstrated that harvesting of BOEC at the degree of expansion used here takes place within a broad and safe window in which genetic instability has not ensued, but acquired endothelial phenotypes would have disappeared [37].

### Microarray method

Sample preparation utilized BOEC trizol lysates and was always performed by the same individual and exactly as previously described [37]. For this study we used the Affymetrix U133A chip (Santa Clara, CA, USA). because our extensive prior validation experiments were performed using it. Biotin-labeled cRNA fragment samples were turned over immediately to our Microarray Core Facility which then returned raw data to us for analysis. To eliminate a source of variability, we purchased one single lot of chips in sufficient quantity for all samples from this project, so there would be no effects due to different lots of chips. We used the robust multiarray average (RMA) method to summarize expression values [42]. Data were background-adjusted, quantile-normalized (global scaling), and expression measures were summarized based on log-transformed perfect match values using median Polish algorithm. Then, we used locally weighted scatter-plot smoothing (LOWESS) to perform within-array normalization [43]. RMA and LOWESS smoothing were implemented in the software Genedata Expressionist Pro3.1PP (Basal, Switzerland).

Microarray data from this study have been deposited at the Gene Expression Omnibus, National Center for Biotechnology Information: GEO accession GSE22688, http://www.ncbi.nlm.nih.gov/geo/query/acc.cgi?acc=GSE22688).

### Analysis of single gene expression

To test for differential expression of single genes, we utilized three approaches. (a) The Welch t-test does not require that variances of expression levels between two groups are equal, but it does assume that they are normally distributed [44]. The resulting *P-*value needs to be corrected for multiple comparisons, but a full Bonferroni correction is believed to be far too stringent for application to microarray data. (b) Significance Analysis of Microarray (SAM) is a nonparametric statistical procedure that employs False Discovery Rate (FDR), and it estimates FDR as an expression of false positive rate [45]. FDR is advantageous because it already adjusts for multiple hypotheses testing [46]. In this study, transcripts with FDR <.05 were considered to be significantly differentially expressed. However, to filter for a smaller field of transcripts, the results we focus on here used the much more highly stringent requirement that FDR = 0. (c) In addition, we simply examined the fold change of single gene expression for AA versus CA subject groups. All analyses of single gene expression were carried out in the statistical software R [47]. Specifically, we used the R function t-test for the Welch t-test and the R package samr for the SAM and fold change analyses.

### Positive internal control and power calculation for analysis of single gene expression

As a positive internal control, we tested the 20 male and 18 female subjects for expression of selected genes. For the male group, 11/11 Y-linked genes showed significantly higher expression in the BOEC from male donors (FDR = 0). For the female group, 17/17 X-linked genes showed significantly higher expression in the BOEC from female donors (FDR = 0). Thus, SAM analysis robustly provided the correct answer.

To estimate the power of this study to detect differential expression of single genes, we performed a power calculation, assuming the requirement for a significance was *P *= 10^-5^, equivalent to *P *= .05 corrected for 5,000 multiple comparisons. The software Java Applets for Power and Sample Size [48] was employed for power analysis for two-sample t-test. Microarray data from 27 BOEC samples from a previous study [37] were used to obtain the expected levels of expression and variance for this estimate. This estimated the power for detecting 1.5-fold differences to be 100% for transcripts at all quartile levels of expression. Power for detecting a 1.25-fold difference was (from highest to lowest expression levels): 100%, 90.5%, 64.1%, 32.9% and 67.8%.

### Analysis of biological systems differences

Examination for differences between study groups in terms of biological systems pattern employed predetermined gene sets. Their construction took place prior to onset of this study, as described elsewhere in greater detail [37,43], and was based on a variety of databases, as well as our review of the relevant literatures. These gene sets are not mutually exclusive, as we wanted them to broadly survey each given biological system, and these systems overlap biologically. For example, the shear stress responsive set includes members of multiple other biologies but which have in common shear responsiveness (see Discussion). The biological systems (number of members in a gene set) were: adhesion (146), angiogenesis (131), apoptosis (79), coagulation (152), hypoxia response (109), inflammation (117), redox signaling (83), shear stress response (156), and vasoregulation (106). The precise composition of these system-oriented gene sets is available elsewhere [49].

To determine whether pre-defined gene sets identified significant biological system differences between AA and CA, we utilized Gene Set Enrichment Analysis (GSEA). This test ranks individual genes in the set on the basis of t-statistic, examines for enrichment (in direction of either higher or lower expression) concordant with subject group assignment, and conducts many permutations to estimate the empirical nominal *P*-value and the FDR q-value, an expression of false positivity likelihood [50]. A gene set with FDR <.25 was considered significant, as suggested by the method's originators, and we tested our dataset in GSEA Java desktop software V2.0 which was downloaded from the authors' website [51].

### Negative internal control and power estimate for analysis by GSEA

As a (potential) negative internal control for the GSEA analysis of the nine biological systems, we utilized gender as a virtual biological system and compared the 20 males to the 18 females. As expected, neither *P-*value nor estimated FDR were significantly different for any of the nine systems for these two groupings (data not shown).

Since there is no (known) way to perform a power calculation for GSEA analysis, we conducted multiple simulations in the statistical software R [47], based on the effect size and variance estimates from 27 BOEC samples from a previous study [37]. Simulation-based power estimation [52] is a generic approach and suitable for non-standard statistical techniques, such as GSEA. These allowed us to estimate that power of identifying significant changes for our pre-determined biological system gene sets (that is, *P *< .05 and FDR <.25) in this study approaches 100%.

### qRT-PCR

This was used to verify gene expression by BOEC for authentic *PSPH *and the PSP-homolog *(PSPHL)*, *SOS1*, *AMFR*, *HSGP25L2G*, *Cxorf12*, and *EIF4B*. These were chosen for qRT-PCR analysis because of their rank in the fold-change assessment for single gene differences. Primers and probes are displayed in Additional File 1. Methods were standard and were performed by the same individual, as previously described [37].

### Phosphoserine phosphatase activity

Previously cryopreserved BOEC from all of the subjects were re-established in culture. BOEC at 90% confluence were harvested for whole cell lysate preparation in phosphate-free buffer using three freeze-thaw cycles. L-phosphoserine phosphatase activities were measured by the release of inorganic phosphate (P_i_) from L-phosphoserine, using an assay somewhat modified from that of Cho *et al. *[53]. The method is described in greater detail in Additional File 2. The phosphatase activity of each sample was expressed as μmol P_i_/μg protein in total cell lysate. We utilized the same method to assess *in vitro *activity against substrate L-phosphoserine of authentic PSP (obtained from abcam, Cambridge, MA, USA) and PSP-homolog (synthesized in the Oligonucleotide and Protein Synthesis Facility in our Biomedical Genetics Center core facility).

## Results

### Single gene expression

#### Analysis by FDR-based SAM

Comparison of BOEC from AA versus CA demonstrated that 31 genes were differentially expressed by the two groups when we employed the highly stringent requirement that FDR = 0. As shown in Table 1, the expression of four genes was increased in AA compared to CA, while expression of 27 genes was lower in AA. (Note that entries in Table 1 are ranked in order of numerical fold-change, so those with the greatest up-regulation are at the top, but those with the greatest down-regulation are at the bottom.)

**Table 1:** Single gene differences (at FDR = 0) by BOEC from AA (n = 21) versus CA (n = 17).*

Affymetrix ID	Gene Name	FDR	Fold Change (AA vs CA)	Welch t-test (*P*)	Gene Description
205048_s_at	*"PSPH" *^*‡*^	0	4.08	4 × 10^-9^	Phosphoserine Phosphatase
212777_at	*SOS1*	0	1.36	1 × 10^-7^	Son of Sevenless Homolog 1
202203_s_at	*AMFR*	0	1.72	4 × 10^-6^	Autocrine Motility Factor Receptor
204379_s_at	*FGFR3*	0	1.24	2 × 10^-5^	Fibroblast Growth Factor Receptor 3
219417_s_at	*FLJ20014*	0	0.91	6 × 10^-5^	Chromosome 17 Open Reading Frame 59
208416_s_at	*SPTB*	0	0.91	1.9 × 10^-4^	Spectrin, Beta, Erythrocytic
206763_at	*FKBP6*	0	0.90	6 × 10^-5^	FK506 Binding Partner 6, 36 kDa
202253_s_at	*DNM2*	0	0.90	2.3 × 10^-4^	Dynamin 2
214380_at	*PRPF31*	0	0.90	9 × 10^-5^	Precursor mRNA-processing Factor 31, S. Cerevisiae, Homolog of
213209_at	*TAF6L*	0	0.89	8 × 10^-7^	TAF6-like RNA Polymerase II, p300/6CBP Associated Factor (PCAF), Associated factor, p65
216204_at	*ARVCF*	0	0.89	4 × 10^-6^	Armadillo Repeat Gene Deletes in Velocardiofacial Syndrome
213585_s_at	*PDCD2*	0	0.89	3 × 10^-5^	Programmed Cell Death 2
201572_x_at	*DCTD*	0	0.89	2.1 × 10^-4^	Deoxycytidylate Deaminase
203589_s_at	*TFDP2*	0	0.89	3.3 × 10^-4^	Transcription Factor DP-2 (E2F Dimerization Partner 2)
211096_at	*PBX2*	0	0.88	3 × 10^-6^	Pre B-cell Leukemia Transcription Factor 2
215844_at	*TNPO2*	0	0.88	8 × 10^-5^	Transportin 2
206182_at	*ZNF134*	0	0.88	2.3 × 10^-4^	Zinc Finger Protein 134
219125_s_at	*LOC55974*	0	0.87	2 × 10^-5^	Stromal Cell Protein
201557_at	*VAMP2*	0	0.87	6 × 10^-5^	Vesicle-associated Membrane Protein 2 (Synaptobrevin2)
202700_s_at	*KIAA0792*	0	0.87	1.3 × 10^-4^	Transmembrane Protein 63A
209009_at	*ESD*	0	0.87	2 × 10^-4^	Esterase D
200076_s_at	*MGC2749*	0	0.86	3 × 10^-5^	Hypothetical Protein MGC2749
207415_at	*PLA2R1*	0	0.86	2 × 10^-5^	Phospholipase A2 Receptor 1, 180 kDa
222129_at	*C2orf17*	0	0.86	1.6 × 10^-4^	Chromosome X Open Reading Frame 17
202646_s_at	*D1S155E*	0	0.85	1.1 × 10^-4^	Cold-shock Domain-containing E1, RNA-binding; CSDE1
222199_s_at	*BIN3*	0	0.85	7 × 10^-5^	Bridging Integrator 3
219939_s_at	*D1S155E*	0	0.82	1 × 10^-4^	Cold-shock Domain-containing E1, RNA-binding; CSDE1
203538_at	*CAMLG*	0	0.82	7 × 10^-4^	Calcium-modulating Cyclophilin Ligand
208757_at	*HSGP25L2G*	0	0.81	1 × 10^-6^	GP25L2 Protein
218354_at	*LOC51693*	0	0.81	3.5 × 10^-4^	Hematopoietic Stem/progenitor Cells 176
204340_at	*CXorf12*	0	0.77	2 × 10^-4^	Chromosome X Open Reading Frame 12
219599_at	*EIF4B*	0	0.76	4 × 10^-5^	Eukaryotic Translation Initiation Factor 4B

*This table shows only those genes that meet the very high stringency level of FDR = 0 by SAM (Significance Analysis of Microarray). Genes are ranked by fold change (AA versus CA) so that those with the greatest up-regulation are at top of the list, but those with the greatest down-regulation are at the bottom. All transcripts exhibiting higher expression in AA versus CA subjects, using the less stringent significance threshold of FDR <.05, are shown in Additional File 3 (n = 21 probe sets, representing 20 genes). Likewise, all transcripts exhibiting lower expression in AA versus CA subjects at FDR <.05 are shown in Additional File 4 (n = 200 probe sets, representing 184 genes).

**Figure 1:**
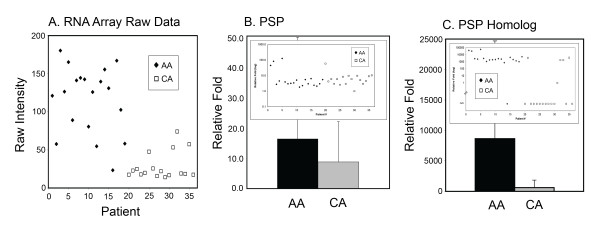
**BOEC expression of *PSPH*, authentic-PSP (*PSPH*), and PSH-homolog (*PSPHL*)**. *PSPH *is the proper gene designation for the L-phosphoserine-phosphatase gene. Its expression per the Affymetrix U133A chip (probest 205048_s_at) seems to show elevation for AA (panel **A**). However, when authentic *PSPH *is measured by qRT-PCR, no difference for between groups is found (panel **B**). Rather, the elevation detected by microarray (panel A) is fully accounted for by increased expression (*P *= .015) of the PSP-homolog, *PSPHL*, in AA >CA (panel **C**). By qRT-PCR, expression of *PSH *for 1/21 AA and for 12/17 CA samples was undetectable.

^*‡ *^This gene designation from Affymetrix is incorrect - see Figure 1

Using the more customary, and less stringent, filter requiring only that FDR <.05, we identified for AA compared to CA: 21 transcripts representing 20 up-regulated genes, and 200 transcripts representing 184 down-regulated genes (see Additional File 3 and Additional File 4, respectively).

#### Analysis by t-test

Each of these Tables additionally displays the results of testing for significance using the Welch t-test. As shown in Table 1 for genes with FDR = 0, the same 4 genes identified by FDR for AA > CA, and 15 of the genes identified for AA < CA, additionally have P < 10^-4^. Since different investigators prefer different stringencies of Bonferroni correction, we display the raw *P*-values.

#### Analysis by fold-change

Fold-change in expression (expressed as AA/CA) is also shown in each Table. In Table 1 the four up-regulated genes with fold-change approximately 1.25 or greater (*PSPH, SOS1, AMFR, and FGFR3*) and the two strongest, equivalently (fold change ≤.80) down-regulated genes (*EIF4B, CXorf12*) are most noteworthy.

### Phosphoserine phosphatase, Affymetrix "*PSPH"*

Of the genes shown in Table 1, "*PSPH" *stands out because it exhibits the highest differential expression among AA subjects (4.08-fold increase, FDR = 0, *P *= 4 × 10^**-9**^) (Figure 1A). However, only two of the Affymetrix probes for *PSPH *are specific for authentic *PSPH*, while the others additionally detect the known homolog, *PSPHL*. Indeed, qRT-PCR revealed that expression of authentic *PSPH *was not significantly different for AA versus CA, and it was very modest (Figure 1B). In contrast, expression of *PSPHL *was significantly elevated (*P *= .015) for the AA subjects (Figure 1C), and this entirely accounts for the false positive array result for *PSPH*. Interestingly, qRT-PCR analysis showed that *PSPHL *was undetectable in 12/17 CA donors but only 1/21 AA subjects.

The homolog, *PSPHL*, has partial sequence identity to *PSPH*, and the 31-residue overlap region contains some, but not all, of those involved in substrate hydrolysis [54,55]. The *PSPHL *product is thus unlikely to display enzymatic activity. Consistent with published data [54], authentic PSP exhibited half-activity on L-phosphoserine at 2 μM, while the PSP-homolog had no activity. To screen for possible inhibitory activity by the homolog, we examined the L-phosphoserine phosphatase activity of AA versus CA BOEC. Despite *PSPHL *having higher levels in AA BOEC (Figure 1C), the L-phosphoserine phosphatase activities of BOEC from AA and CA were equivalent: 1.16 ± 0.22 and 1.21 ± 0.31 μmole P_i_/μg total cell lysate, respectively. The amount of endogenous free P_i _in AA BOEC (2.80 ± 0.77 μmole P_i_/μg) was slightly, but not significantly, higher than in CA BOEC (2.29 ± 0.93 μmole P_i_/μg).

### qRT-PCR

We used qRT-PCR to examine (previously-cryopreserved) BOEC from all study subjects for expression of several genes from Table 1, and fold-changes were 1.26 for authentic-*PSPH *(*P *= NS) and 3.96 for *PSPHL *(*P *= .0154) for AA versus CA. As predicted from the small fold-changes detected by microarray, qRT-PCR was unable to confirm a significant difference for the generally similar fold-changes for *SOS1*, *AMFR*, *HSGP25L2G*, *Cxorf12*, or *EIF4B*.

### Biological systems analysis

To consider whole biological systems relevant to endothelial biology, we used GSEA to test BOEC from AA versus CA for evidence that any of nine specific systems tended to have altered expression: adhesion, apoptosis, angiogenesis, coagulation, hypoxia response, inflammatory signaling, redox response, shear stress response, and vasoregulation [37,49]. This comparison revealed a significant whole-system differential expression in one biological system, shear stress response genes. This was evidenced not only by *P *= .027 and FDR = .14, but also by the fact that these indicators are, simply by inspection, unambiguously different from the values for the other eight systems (Table 2). The component composition of the shear response gene set is provided in Table 3 which also indicates which members of this set actually contributed to identification of this particular biological system as being different for AA versus CA (that is, those exhibiting "core enrichment"). The expanded names of these gene identifiers are provided in Additional File 5. All genes within the core enrichment group were found to have changed in direction of increased expression.

**Table 2:** GSEA results for nine biological systems, comparing AA versus CA.*

Biological System	Gene Set Size	Nominal *P*	FDR
Adhesion	146	0.907	.100
Angiogenesis	131	0.960	.946
Apoptosis	79	0.202	.551
Coagulation	152	0.416	.815
Hypoxia	104	0.547	.780
Inflammation	117	0.989	.987
Redox	83	0.788	.932
Shear stress	156	0.027	.140
Vasoregulation	101	0.822	.920

* For GSEA (Gene Set Enrichment Analysis), FDR <.25 is considered to be significant (see Methods). Construction and specific composition of gene sets has been presented elsewhere [37,49].

**Table 3:** Shear Stress Biological Gene Set (156 Members)

Gene Contributes to Core Enrichment		Gene Does Not Contribute to Core Enrichment		
BMP4	SP1	ACTB	GJA1	PDPK1
BMP6	TBXAS1	AKAP1	GNAS	PECAM1
CCL2	TGFB1	ANXA2	GNB2	PFDN5
*CD34*	*THBD*	*ANXA5*	*GNG5*	*PTGS2*
*CENPF*	*THBS1*	*APOE*	*GRN*	*RGS5*
*CTGF*	*TRA1*	*APS*	*HADHSC*	*RPL30*
*CXCL12*	*TUBG1*	*ARF4L*	*HMOX1*	*RPL34*
*CYP1B1*	*VCAM1*	*ASS*	*ICAM1*	*RPS11*
*CYR61*	*VIPR1*	*CAV1*	*IFITM3*	*RPS7*
*DPYSL3*	*XPO1*	*CCL15*	*IL13RA1*	*SAT*
*EDN1*		*CCL25*	*IL15*	*SCGF*
*EEF1A2*		*CD164*	*IL16*	*SELE*
*ELN*		*CD1D*	*IL1R1*	*SERPINE1*
*F3*		*CD58*	*IL1RL1*	*SERPINE2*
*FGF2*		*CD68*	*ITGB3*	*SLC35F2*
*FN1*		*CDKN1A*	*JARID1A*	*SMARCD1*
*GAPD*		*CEACAM1*	*JUN*	*SOD2*
*GBP1*		*CYP1A1*	*KLF2*	*SPARC*
*GJA5*		*DKK2*	*LAMB1*	*SPTA1*
*GSTP1*		*E2F5*	*LIMS1*	*SPTAN1*
*IL8*		*EDN3*	*LRP2*	*STAM*
*ILK*		*EFEMP1*	*METAP2*	*TEK*
*JAG2*		*EGR1*	*MGP*	*TFPI*
*JUNB*		*EIF4EBP2*	*MMP1*	*TGFB1I4*
*KIF20A*		*EIF4G3*	*MMP14*	*THBS4*
*KLF4*		*ESM1*	*MMRN1*	*TNFRSF1A*
*LIG3*		*F2*	*MYD88*	*TNFRSF5*
*MAPRE1*		*F2R*	*NFKB1*	*TNFSF8*
*MATN2*		*FGF6*	*NOTCH4*	*TUBA3*
*MYC*		*FGFR3*	*NQO1*	*TXNRD1*
*NOS3*		*FLT1*	*NUMA1*	*UNG*
*PFDN2*		*FOS*	*OGT*	*VCL*
*PLAT*		*FOSL1*	*OLR1*	*VEGFC*
*RGS3*		*FTH1*	*PBP*	*VWF*
*RHOA*		*FTL*	*PCQAP*	*WNT2B*
*RHOB*		*GARS*	*PDGFRA*	
*S100A10*		*GAS*	*PDGFRB*	

"Core enrichment" indicates that the corresponding gene is in the subset of genes that contributes most significantly to that biological system's result using GSEA (Table 2) in the text. In the table above, entries are listed in alphabetical order. An expanded version of these gene names is available as Additional File 5.

## Discussion

We here examined whether, at the level of gene expression, there are detectable endothelial cell differences associated with continent-of-origin that possibly could impact on the biology of the vessel wall such that risk for vascular disease would be influenced. The present results indicate that the endothelial expression of a number of genes does differ for AA versus CA, and the biological system of shear stress responsiveness is different for AA as well. The high relevance of these observations to endothelial biology is discussed below. But in summary, the present results support the notion that genetically-determined differences in endothelial gene expression (determined by ancestral continent of origin) can influence vascular wall biology and, therefore, impact on vascular disease risk. Moreover, this study illustrates the feasibility of utilizing this method to help bridge the gap between structural information provided by genomics and the consequent impact upon cell biology and pathophysiology.

To accomplish our goal we took advantage of a unique source of endothelial reporter cells, BOEC, which have never been influenced by *in vivo *signalling, for example, by the plasma milieu or tissue-specific factors. Therefore, their phenotype is influenced only by culture conditions and the genetic composition of the BOEC donor. Since the cultures are performed meticulously and are controlled fastidiously, we believe that this method can preliminarily identify potential, genetically-influenced differences in endothelial cell biology. A previously reported example of this approach is noted in the Introduction, and it describes the methods validation that was conducted for both BOEC cultures and specific application of microarray profiling to them [37]. Since BOEC, unlike other endothelial sources, cryopreserve well they can be banked at time of initial culture harvest and be saved for confirmatory future examination by cell biologic or other methods in studies that have been informed by the gene expression results.

### Single gene expression

We detected 31 genes that met our most stringent criterion for differential expression significance, that FDR = 0, with 4 changed in the AA > CA direction, and 27 changed in the AA <CA direction (Table 1). All transcripts that showed a significant degree of change at the much more commonly utilized threshold of FDR <.05 are displayed in Additional File 3 (21 transcripts for 20 genes for AA >CA) and Additional File 4 (200 transcripts for 184 genes for AA <CA).

#### *PSPH *and *PSPHL*

"*PSPH*" was differentially expressed (AA >CA) at highest fold-change. However, this was fully accounted for by elevated expression of the known homolog, *PSPHL *(Figure 1), which exhibited no activity either as a L-phosphoserine phosphatase or as an inhibitor thereof. No studies have examined it for the possibility of other biological activities. Nonetheless, since its expression distributes largely according to self-identification of race (Figure 1), it conceivably is of interest. It may be useful as a marker of continent-of-origin, and this is the likely reason that increased *PSPHL *expression has been noted in prostate and breast cancers of AA individuals, as well as specifically in the tumor stroma of breast cancer [56,57]. Yet, since most CA (12 of 17) in the present study had no detectable expression of the homolog, the (unknown) reason that its expression was detectable at all in most AA (20 of 21) might itself be of interest. To date, the cell-type restriction, if any, for its expression has not been reported. It is known that *PSPH *and *PSPHL *reside at different locations: chromosomes 7p 15.1 to 15.2 and 7q 11.2, respectively.

As a caution to investigators, we note that the "*PSPH*" Affymetrix 205048_s_at probeset (on both U133A and U133A 2.0 arrays) actually detects both *PSPH *and *PSPHL*, as described above. This same probeset has erroneously been labelled *PSPHL *in at least one previous report [56], and is erroneously labelled *PSPH *by Affymetrix.

### Other genes of possible interest

Many of the genes shown to be differentially expressed at the thresholds of FDR = 0 (Table 1) and FDR <.05 (Additional Files 3 and 4) have direct relevance to endothelial cell biology. While we do not wish to overstate their potential importance, the following discussion of some from Table 1 (presented in order of their listing therein) attempts to illustrate possible biological roles.

#### SOS1

(son of sevenless homolog 1) is a necessary factor in transduction of angiopoietin I signaling-induced chemokinesis of endothelial cells [58]. AA men and women tend to initially present with more advanced stages of prostate and breast cancer [56,57]. Therefore, the possibility that elevated endothelial expression of *SOS1*, as recently found in breast tumor mixed-cell stroma in AA women [57], could contribute to more exuberant angiogenesis (see below), critical to tumor growth [59], should perhaps be tested.

#### AMFR

(autocrine motility factor receptor) is involved in angiogenesis, endothelial motility, and increased permeability; and its secretion by tumor cells is reported to up-regulate the vascular endothelial growth receptor Flk-1 [60]. It too showed increased expression in prostate cancers in AA [56], and a role similar to *SOS1 *can be considered.

#### FGFR3

(fibroblast growth factor receptor 3) is induced in lymphatic endothelium by its developmental "master switch" Prox1, and it plays a role in the biology of lymphatics which are important in inflammation and tumor biology [61]. AA reportedly present with more aggressive forms of, for example, breast and prostate cancer [57,62].

#### DNM2

(dynamin 2) is a GTPase that activates eNOS, is a critical regulator of vascular tension, and may participate in signaling by one of the vascular endothelial growth receptors [63,64]. Diminished activity of eNOS among AA would, of course, have substantial implications for endothelial function and vascular biology (see Introduction).

#### TAF6L

(TAF-like RNA polymerase II, PCAF, p65) modulates chromatin structure via association with histone acetylase [65] and is a common cis-element in protein synthesis and cell cycle promoters [66].

#### ARVCF

(Armadillo repeat gene deletes in velocardiofacial syndrome) is ubiquitously expressed, and it associates with cadherins at junctions between cells [67]. Although binding to the endothelial cadherin (VE-cadherin) is within its repertoire, its function has not been specifically examined in endothelial cells; so it is difficult to predict what effect its diminished expression might have in these cells.

#### BIN3

(bridging integrator 3) is involved in actin localization and signalling [68], and it is ubiquitously expressed. Although an endothelial role *per se *for *BIN3 *has not been tested, actin plays a role in endothelial mechanotransduction. Hence, its diminished expression in BOEC from AA perhaps warrants study, given the altered expression of shear responsive genes that we observed in the biological systems approach (see below).

#### CAMLG

(calcium modulating ligand) is involved in angiotensin II (AGNII) signaling, and *CAMLG *interaction with AGNII-type I receptor is suggested to be a participant in ANGII vasoregulatory actions [69]. This, of course, is highly relevant to hypertension.

#### EIFB4

(Eukaryotic translation initiation factor 4B) is a cofactor in initiation of protein translation. It is unknown whether its own diminished expression might conspire with the unrelated diminished transcription observed here of multiple other genes among AA (Additional File 4) to impact on endothelial biology by augmenting translational inefficiency. However, since this presumably would adversely impact the endothelial cell's responses to stress such as inflammation or shear, it seems worth considering.

### eNOS

Our results revealed no significant difference between our study groups in *NOS3 *expression: AA >CA by 1.31-fold; t-test *P *= .071; FDR = not significant. Thus, the eNOS-related differences observed both clinically and experimentally (see Introduction) perhaps reflect acquired changes (e.g., enzyme uncoupling) rather than alterations in the endothelial expression level of the protein. As noted above, one such possibility would perhaps derive from the diminished level of *DNM2 *presented above.

### Biological systems approach

Our second analysis examined whether any of nine biological systems relevant to endothelial cell biology would display a tendency toward altered expression for AA versus CA. Indeed, on a systems basis, AA had significantly altered expression of the gene set representing the shear response biological system, a major determinant of endothelial phenotype and vascular homeostasis (Table 2). The component members within this gene set which contributed to this result are shown in Table 3.

**Figure 2:**
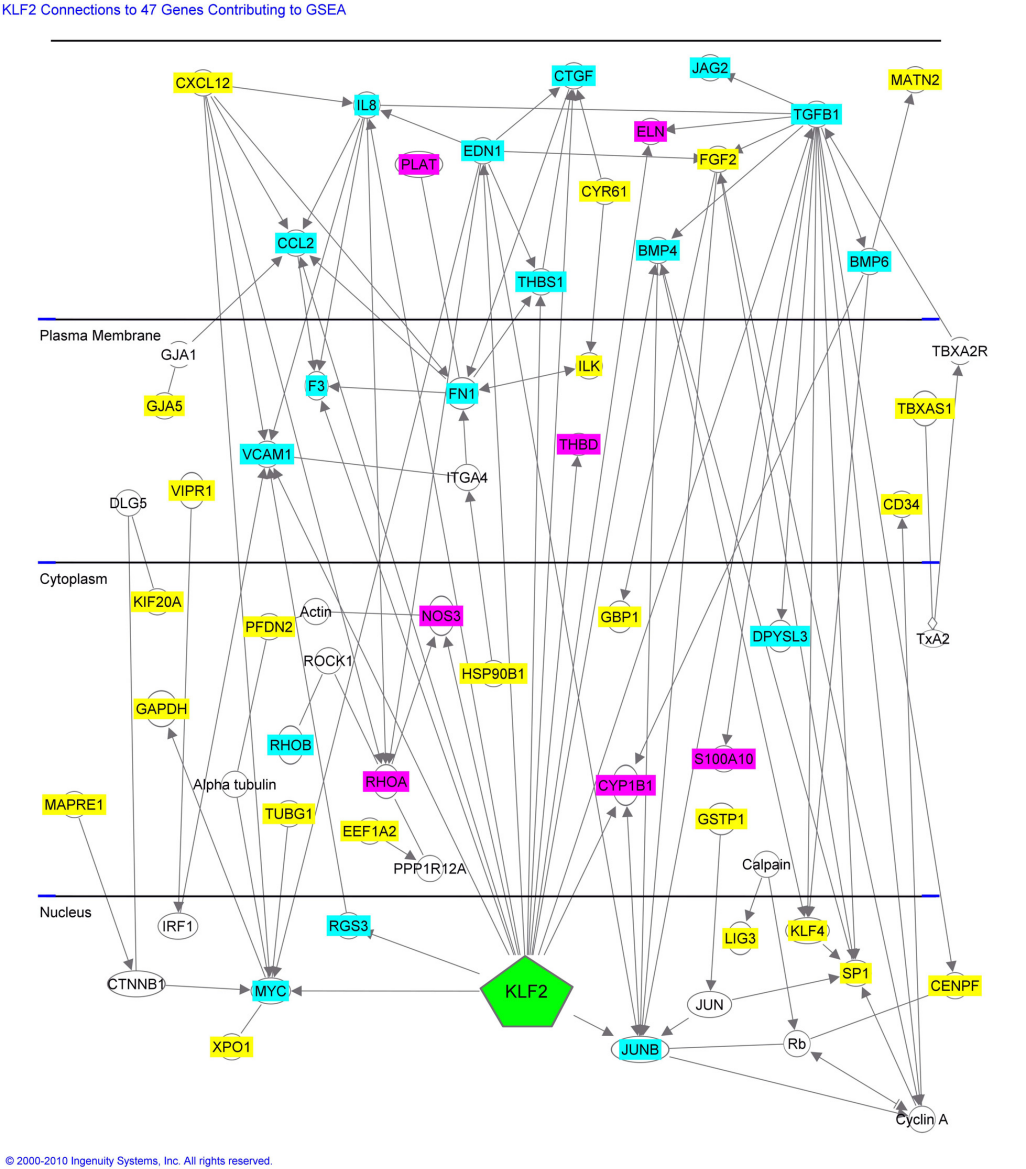
**KLF2 connections to 47 genes contributing to shear responsive biological system result**. The relationship of transcriptional regulator KLF2 to each of the 47 genes contributing to the difference in the shear responsiveness biological system is shown here. KLF2 up-regulates genes shown in magenta and down-regulates those shown in blue. Genes shown in yellow are not directly regulated by KLF2. Genes shown in white are not members of the shear responsive gene set but are linkers extant in the displayed relationships. This figure was constructed via access to Ingenuity Systems, Inc.^® ^[87]

Laminar shear stress induces endothelial "quiescence" (anti-inflammatory, anti-thrombotic, anti-adhesive, anti-angiogenic, anti-oxidant, anti-atherosclerotic, and with optimal vasoregulatory balance), while areas of low or disturbed shear stress exhibit opposite changes [70-73]. This actually is oversimplified, since experimental data indicate that the induced results also can differ depending upon whether the flow is pulsatile or oscillatory. In any case, shear responsiveness is mediated partly by its induced levels of transcription factor KLF2 (Kruppel-like factor-2), a "master switch" that regulates perhaps a third of the approximately 1,000 shear responsive genes. For example, the 47/156 members of the shear responsiveness gene set that accounted for the observed difference between AA and CA are depicted in Figure 2, which additionally highlights those that are directly influenced by KLF2. The genes that share the property of shear responsiveness actually are participants in multiple endothelial cell biologies as is summarized in limited fashion in Table 4. For example, some of the chemokines that promote development of atherosclerotic lesions are shear responsive [74], for example, CCL2 (monocyte chemoattractant protein 1) which is one of the genes contributing to the present implication of shear responsiveness (Table 3). Notably, KLF2 and NFκB down-regulate each other as significant regulators in orchestrating endothelial inflammatory phenotype.

**Table 4:** Shear stress biology genes contributing to core enrichment: spectrum of vascular biological functions

	Cell Migration	Angiogenesis and Vascularization	Vasomotor	Immune System	Chemotaxis, and so on	Hemostasis and Hematologic
**Gene name**						

*BMP4*	X	X	X	X	X	

*BMP6*			X			X

*CCL2*	X	X		X	X	

*CD34*	X			X	X	

*CENPF*						

*CTGF*	X	X				

*CXCL12*	X	X			X	

*CYP1B1*		X	X			

*CYR61*	X	X	X		X	

*DPYSL3*	X					

*EDN1*	X	X	X	X		

*EEF1A2*						

*ELN*			X		X	

*F3*	X	X			X	X

*FGF2*	X	X			X	

*FN1*	X	X	X		X	X

*GAPD*		X			X	

*GBP1*						

*GJA5*	X	X			X	

*GSTP1*						

*IL8*	X	X		X	X	X

*ILK*	X	X			X	X

*JAG2*	X			X		

*JUNB*		X			X	

*KIF20A*						

*KLF4*		X		X		X

*LIG3*						

*MAPRE1*						

*MATN2*	X					

*MYC*	X	X		X	X	

*NOS3*	X	X	X	X	X	

*PFDN2*						

*PLAT*	X	X			X	X

*RGS3*	X				X	

*RHOA*	X	X			X	X

*RHOB*					X	

*S100A10*		X			X	X

*SP1*		X		X	X	X

*TBXAS1*	X	X	X			X

*TGFB1*	X	X		X		

*THBD*				X		X

*THBS1*	X	X		X	X	X

*TRA1*				X		

*TUBG1*						

*VCAM1*	X			X	X	

*VIPR1*					X	

*XPO1*						

The present study cannot identify whether the changes observed for AA would yield a net beneficial or net harmful effect on vessel wall biology, or whether they would even alter biological shear responsiveness. The increased expression among AA was inexplicably observed for genes that, in the normal process of establishing a quiescent endothelium, both increase in response to KLF2 (for example, *NOS3*, *PLAT*, *THBD*) and decrease in response to it (for example, *EDN1*, *F3*, *TGFB1, JUNB*, *VCAM1*) (Table 3, Figure 2). The explanation for this is not known at this time. Interestingly, there was no concurrent differential expression of the two major shear response "master switches," *KLF2 *(fold-change 1.05, *P *= .646, FDR = .678) or *Nrf2 *(1.06-fold change, *P *= .408, FDR = .588). However, we emphasize that the present method would be insensitive to, for example, a *KLF2 *SNP that alters its function but not expression.

Yet, this dilemma may derive, in part, from the fact that BOEC in this study perhaps would have had a relaxed shear response system, as opposed to a shear-modulated one. Unfortunately, existing shear responsiveness studies are not very enlightening regarding this concept because they generally have described changes induced by a shear regime rather than possible inter-individual differences in the starting phenotype. We speculate that the present data, in suggesting a tonic increase in multiple shear responsive genes for AA subjects, may be hinting at some underlying, functional variation of the apparatus that senses shear stress and induces phenotypic changes. We emphasize, however, that there is no direct evidence for this at the present time. Shear responsiveness is complex, incompletely understood, and involves multiple steps: mechanosensitivity, trans-membrane mechanotransduction, chemical mechanotransduction, downstream signalling and feedback loops, and ultimately transcription and translation [70]. We are currently utilizing the banked, cryopreserved BOEC from the AA and CA subjects in the present study to examine their gene expression shear responsiveness in a flow chamber system, and also to further delineate reason(s) for any difference.

### Integration of results and disease biology

The importance of the endothelial cell in disease biology cannot be overstated. It resides at the physical and functional interface between blood and tissue and somehow integrates the input from a panoply of biological modifiers. Some of the physiological processes regulated by these cells are noted in the Introduction. Beyond the conclusions and speculations already presented, we cannot say exactly how the observed gene expression changes, if present *in vivo *in AA, actually impact upon vascular biology. However, the potential ways in which genetically-influenced heterogeneities in endothelial biology could impact on disease genesis are endless. The present implication of shear responsive genes is particularly interesting, given the fundamental role that this biological system plays in vascular homeostasis. In particular, it is directly relevant to the problem of hypertension in AA, the disease process that is the most likely candidate to reside proximate to the other vascular problems. Interestingly, this biological system is particularly amenable to influence by gene-environment interaction. Its functional integrity is susceptible to the confluence of effects of salt, lipids, arterial wall stiffening, oxidation biology, endothelial dysfunction and disturbance of vasodilators and vasoconstrictors.

Specific examples of dietary salt and lipid influences were noted in the Introduction. Emerging data emphasize the common occurrence of cardiovascular disease despite an absence of customary risk factors and suggest the importance of coincidental concurrence of genetic variability (genetic high risk) and environmental factors such as tobacco smoke exposure (environmental high risk) in premature and/or accelerated disease genesis [30]. An illustrative example of this in the general population, inherited variation in *APOE *alleles, is provided by the large role that oxidative stress probably plays in cardiovascular disease genesis. Compared to the majority of individuals having the ε3 isoform (gene frequency approximately 0.77), the fewer individuals having the ε2 isoform (gene frequency approximately 0.08) have the highest levels of blood APOE and lower cholesterol levels and protection from cardiovascular disease and stroke. Conversely, those with the ε4 isoform (gene frequency approximately 0.15) have the lowest APOE levels and higher cholesterol. However, the disease risk elevation observed for ε4 individuals is largely seen in those having environmental exposure to tobacco smoke [30]. Furthermore, the antioxidant capability of APOE shows apoE2 > apoE3 >apoE4, and the latter individuals exhibit higher levels of lipid hydroperoxides, which are harmful to endothelium [30]. In linking genetic makeup with dietary habits and environmental exposure, this provides a dramatic example of gene-environment interaction and the contextual complexity of its role as a determinant of vascular disease phenotype. As noted elsewhere: *'Genes load the gun, but the environment pulls the trigger' *(attributed to Dr. Elliot Joslin) [30].

Finally, as noted in the Introduction, health disparities occur worldwide and in many forms. Undoubtedly, some reflect mostly environmental/social factors and others are more dependent upon genetic factors. However, a genetic admixture at some level is a universal characteristic of the human population, so the present study is widely relevant in the general sense. Admixture mapping studies for disease association loci are in the earlier stages of development, so at this time they are applied to more dramatic cases of genetic admixture in populations. One example, derived from an admixture a few hundred years (approximately six to seven generations) ago, is described here. Another prominent admixture occurred in western China, where the Uyghurs display an approximately 50/50 admixture of Asian and European backgrounds [75]. This example occurred more remotely, perhaps approximately 100 generations ago, and is likely explained by the fact that the Uyghurs have lived astride the historical Silk Road. Therefore, it most likely represents a version of admixture that is more historically representative of the human experience, admixture accompanying commerce or migration.

### The endothelium in cancer biology

Quite separately from cardiovascular health, endothelial cells play a fundamental role in cancer biology since tumor growth is rate-dependent on angiogenesis [59]. AA are known to have cancers (for example, of prostate in men [56,62] and breast in women [57,76]) that exhibit greater aggressiveness, present in more advanced stages, and entail higher mortality rates, compared to their behavior in CA. Of great relevance to the theme of the present study, an examination of breast tumor microenvironment suggested that it exhibits a greater degree of angiogenesis (largely a host response to tumor presence) in AA compared to CA [57]. And in the general population, increased likelihood of invasive and metastatic breast cancer (previously shown to be influenced by the degree of tumor angiogenesis) is associated with specific vascular endothelial growth factor alleles [76], as is the aggressiveness of prostate cancer in men [62]. Therefore, the approach used in the present study may well be of use to further identify the influences of genetics and continent-of-origin on the endothelial biology of angiogenesis insofar as it contributes to cancer cell behavior.

### Caveats regarding this study

#### Self-identification of race and genetic admixture

Race is strictly a societal construct based on awareness of superficial traits, but it has been used for (presumptively) identifying continent-of-origin ever since genetic considerations began being applied to humans. Although self-identification of race is hazardous in societies that have high degrees of both diversity and genetic admixture (for example, Brazil [77]), within the United States excellent correspondence between self-reported race/ethnicity and genetic markers reflecting ancient geographic ancestry has been observed [78].

On the other hand, a genetic admixture of individuals having different ancestral origins is the major determinant of genetic structure in the United States [78]. Thus, an admixture of European and African genetic backgrounds over six to seven generations has resulted in the average African American having approximately 20% European admixture [79,80], although on an individual basis this ranges widely from approximately 5% to approximately 70% [80]. The African origin component is almost entirely from West and West-Central, sub-Saharan Africa [79]. In our region of the United States, the Upper Midwest, reported degrees of admixture are similar, for example, approximately 19% for Chicago and approximately 16% for Detroit [80,81]. The level of admixture in the opposite direction, African into European, is estimated to be about 0.4% [80].

The degree of admixture in AA probably exerts an impact upon development of disease, as revealed by the expanding use of admixture mapping [82]. In turn, one should expect that variation in degree of admixture could exert an influence upon endothelial gene expression, as studied here, and upon endothelial function, as implied by the present studies. However, insofar as African origin accounts for the differences observed here, the effect of admixture on present results would be in the direction of diminishing the difference between the AA and CA. In other words, differences in endothelial gene expression reflecting continent-of-origin are observed here despite any diluting impact of admixture. That the degree of admixture for the present subjects is unknown should not be an issue, since the very point of this study was to examine AA subjects sampled in the same fashion they were for the seminal studies demonstrating that, despite undefined individual degrees of admixture, the AA sub-population has higher level of stroke and hypertension [2,3].

We note, however, that our approach could be productively employed to study the endothelial features of subjects who were specifically selected for their known and defined degree of admixture(s).

#### Absence of detailed medical information

The 20- to 29-year-old subjects selected for this study claimed cardiovascular health, and incidentally were not obese. We did not examine them for risk factors such as BMI, blood lipid levels, fasting glucose, hypertension and so on. However, as for the preceding section, that was the point of this first study. Without pre-study bias due to knowing such information, we obtained the present results. We again note that our approach could be utilized to study subjects known to have specific disease risk variables, and specific perturbing influences of interest (for example, oxidized lipid) could thereby be examined *in vitro *in a known genetic context.

#### Are "young and healthy" subjects really healthy?

Consistent with the preceding paragraphs, the intention of this study was to examine AA who were "young and healthy" in the sense that they would be so judged during a visit to a medical clinic, prior to conduct of any medical history, examination, or testing. Indeed, statistical divergence of the higher AA prevalence of first myocardial infarction (the gold-standard clinical indicator of underlying vascular disease) does not emerge until the 35 to 44 age range [8].

On the other hand, the situation for those <35 years is more an absence of evidence rather than evidence of absence. Indeed, it is notable that onset of some cardiovascular risk conditions prior to adulthood has become a feature of modern society, as evidenced by the epidemic of obesity among children and adolescents in the United States. Compared to CA in the same age group, AA adolescents have a lower prevalence of smoking (approximately 13% versus approximately 26%) but a higher prevalence of an inadequate level of physical activity (approximately 39% versus approximately 30%), and adolescent AA females, but not males, have an increased prevalence of obesity (approximately 24% versus approximately 15%) [83]. In the general adolescent population, obese children are more likely to exhibit higher systolic blood pressure [84] and blood lipid levels [85], and to have type-2 diabetes at 9- and 26-year follow-up visits [86].

Although these data suggest that the "young and healthy" may not actually be so healthy, the correspondence between many of these risk factors is with obesity. We are unable to find in the literature the prevalence of such risk factors for non-obese AA children and adolescents. Nonetheless, the present method offers a way to actually test the endothelial biology of (genetically- or clinically-defined) high-risk adolescents, long before they actually develop clinical cardiovascular disease.

#### Small number of subjects

The present results were obtained by comparison of a relatively small number of subjects (n = 21 AA, and n = 17 CA). However, as noted in Methods, power estimates based on the present and prior data from our laboratory reveal that with about 20 subjects in each of two groups, our method has an approximately 100% power of detecting single gene expression differences if they are 1.5-fold or greater, with erosion of power for lower fold changes. In practice, as is evident here, we have found that the potential sensitivity of the method has somewhat greater power than predicted.

#### Verification

Although we cannot "increase the n" for the present study (since an aspect of the method is use of a single lot of chips and a single batch of culture components), we did seek verification of our present results by examining another data set.

We applied the present GSEA analysis method to a previous (and non-overlapping) group of AA (n = 18) versus CA (n = 9) that were controls for our previous sickle stroke study [37], and we again identified a significant difference for shear stress responsiveness genes: *P *= .033, FDR = .140. However, this significance was weaker than the current one because it was true only for the core enrichment sub-group of the shear responsiveness gene set (that is, the sub-group that actually accounts for the present implication of that biological system (Table 3)). This probably is because the prior subject groups were of less homogeneous age range (18 to 60), and group size was much smaller. Furthermore, subjects for that previous study were recruited from our health center itself, so either or both CA and AA groups could have been enriched for those with established disease (that is, potentially having higher genetic risk profile).

Adding additional support to the present results, we note that for those single genes that exhibited significant differential expression for both previous and current AA versus CA data sets, the correlation between them for the fold-change (AA versus CA) was excellent (r = .810). Finally, we now understand that our preliminary observation in that prior report that shear stress responsiveness did not differ for comparison of AA versus CA [37], was simply because it employed a much less-sensitive (and now discarded) method for analysis. Verifying this, application of that old method to the present study groups also fails to identify the significant AA versus CA differences evident for the present study groups. In aggregate, these considerations provide verification for the present results.

## Conclusions

Using blood outgrowth endothelial cells (BOEC) as reporter cells, we determined whether, at the level of gene expression, there might be insights related to the excess vascular disease burden of AA compared to CA. At the high stringency threshold requiring FDR = 0, this approach successfully identified 31 genes that exhibited differential expression among AA. At the more customary stringency level requiring FDR <.05 considerably more were differentially expressed (221 transcripts representing 204 genes). Many of the identified genes are directly relevant to endothelial cell biology, for example to vasoregulation or angiogenesis. In our separate approach, use of pre-determined gene sets to survey expression within each of nine biological systems relevant to endothelial cell biology revealed an apparent tonic increase for many genes within the biology of shear stress responsiveness, which is a major determinant of endothelial phenotype and vascular homeostasis, including its regulation of vascular tone. Consequently, our results provide support for the concept that inherited inter-individual variation in endothelial gene expression, reflecting continent-of-origin, might impact on endothelial cell and vascular biology and, thereby, upon disparity in vascular and cancer disease burdens for AA compared to CA. Also, our results identify needed research directions.

It is true that the present approach requires participation of subjects that are of known genotype or clinical phenotype (in terms relevant to the specific study question at hand), as well as very meticulous performance of laboratory procedures. Nonetheless, the ability of this method to provide information from small groups of subjects suggests that it may be of substantial value to future studies of genotype-phenotype correlation as the field of functional genomics evolves toward a whole cell, systems biology perspective.

## Abbreviations

AA: African Americans; BOEC: blood outgrowth endothelial cells; CA: Caucasian Americans; FDR: false discovery rate; GSEA: gene set enrichment analysis; *PSPH: *L-phosphoserine phosphatase; *PSPHL: *the PSP-homolog; RMA: robust multiarray average; SAM: significance analysis of microarray.

## Competing interests

The authors declare that they have no competing interests.

## Authors' contributions

PW co-designed the study, and performed all statistical analyses. JE supervised construction of gene sets for the biological systems and performed interpretative liaison functions to link statistical and cell biologic aspects of the study. LCM performed all biochemical and molecular biologic aspects of the study. JN recruited subjects, obtained blood samples, established BOEC cultures, and performed quality control measures. WP co-designed statistical approaches and supervised their application. RPH conceived and co-designed the study, supervised its laboratory aspects, reviewed raw data, and supervised quality control aspects. PW, JE, LCM and RPH jointly wrote the manuscript, and RPH edited its final version and revision.

## Pre-publication history

The pre-publication history for this paper can be accessed here:

http://www.biomedcentral.com/1741-7015/9/2/prepub

## Supplementary Material

Additional file 1 **Primer and probe sets for qRT-PCR**. Click here for file

Additional file 2 **Supplemental method**. Click here for file

Additional file 3 Single gene differences at FDR <.05 stringency level showing AA >CA*. Click here for file

Additional file 4 Single gene differences at FDR <.05 stringency level showing AA <CA*. Click here for file

Additional file 5 **Shear stress biology gene set - expanded gene names**. Click here for file
