# Supplementary material for: Differential endothelial cell gene expression by African Americans versus Caucasian Americans: a possible contribution to health disparity in vascular disease and cancer
Source: BMC Med. 2011 Jan 11;9:2. doi: 10.1186/1741-7015-9-2 (PMC3029215; doi:10.1186/1741-7015-9-2)
Supplement: Additional file 1 — Primer and probe sets for qRT-PCR. [file 1741-7015-9-2-S1.PDF]

Additional File 1
Primer and probe set for qRT-PCR

Gene	Sequence
PSPH	Sense: 5’-GATGCTGTGTGTTTTGATGTTGAC -3’<br>Antisense: 5’-CTTGACTTGTTGCCTGATCACATT -3’<br>Probe: 5’-FAM-CGATTCCTTCTTCACTGATGA-BHSQ-3’
PSP	Sense: 5’- CAAGCTCTGCCTCCCAGGTT -3’<br>Antisense: 5’- GGCGGATCACAAGGTCAGGA -3’<br>Probe: 5’-FAM- CGCCATTCTCCTGCCTCAGCCTCC-TAMRA -3’
PSP Homologue	Sense: 5’- GTTGGGTGTCCGCAGGCTTC -3’<br>Antisense: 5’- CTTCTGCGCTACCTTGCGATC -3’<br>Probe: 5’-FAM-TGCCCGCCACTTCCCGCAGGAC-TAMRA-3’
SOS1	Sense: 5’-ACCAAATTCTCCAAGAACACCGTT-3’<br>Antisense: 5’-GGAGGAGGGACAGGCACTTC-3’<br>Probe: 5'–FAM-CCTCCGCCTGCTTCTGGTGCTTCC-TAMRA-3’
AMFR	Sense: 5’-TCAGGGAAGAACATCAAGGAGAGA-3’<br>Antisense: 5’-TGGTGGTGTGCATCACTTCAAC-3’<br>Probe: 5'–FAM-AACTCGGCAGCCAGCTCGCAATCC-TAMRA-3’
HSGP25L2G (TMED9)	Sense: 5’-GGACCTCCTGGAACTGACTTCTT-3’<br>Antisense: 5’-GCCTTCAGCTTGTGCCTGTG-3’<br>Probe: 5'–FAM-TGCCTCCAGCCCCTCTCCCTTCCA-TAMRA-3’
Cxorf12	Sense: 5’-CACCACCTCGGCTACCTACTT-3’<br>Antisense: 5’-CCTGTGAGGCACTGGAAGAGA-3’<br>Probe: 5'–FAM-CCACCGTGCGAGCTGATGGTCACA-TAMRA-3’
EIF4B	Sense; 5’-TCGGTTTCTTGATGTCTTTTGGTT-3’<br>Antisense: 5’-ACTGTCCCTGTCCCTATTTATCCA-3’<br>Probe: 5'–FAM-TGCCTGCTCCTGATGCTTGGACCC-TAMRA-3’
