# Supplementary material for: Differential endothelial cell gene expression by African Americans versus Caucasian Americans: a possible contribution to health disparity in vascular disease and cancer
Source: BMC Med. 2011 Jan 11;9:2. doi: 10.1186/1741-7015-9-2 (PMC3029215; doi:10.1186/1741-7015-9-2)
Supplement: Additional file 2 — Supplemental method. [file 1741-7015-9-2-S2.PDF]

Additional File 2: Supplemental Method

In brief, the 50 ml of reaction mixture containing 15 mg of whole cell lysate (20 mM Tris-HCl, pH 7.5, 1 mM MgCl2) was incubated at 37o C for one hour, followed by addition of 5 ml of 0.2 M EDTA, and it was placed on ice to stop the reaction. The amount of inorganic phosphate (Pi) released was immediately measured using the EnzCheck Phosphate Assay Kit (Molecular Probes, Eugene, OR) according to the manufacturer’s protocol. Serial dilutions of KH2PO4 (5- 200 mM) were always included for Pi standard. The amount of the endogenous inorganic phosphate in each whole cell lysate was measured directly by using the same Phosphate Assay Kit. Each sample was measured in triplicate. The phosphatase activity in each sample was obtained from the value of whole cell lysate after subtraction of the value of endogenous Pi. The phosphatase activity of each sample was expressed as mmol Pi /mg of total cell lysate.
