# Supplementary material for: Differential endothelial cell gene expression by African Americans versus Caucasian Americans: a possible contribution to health disparity in vascular disease and cancer
Source: BMC Med. 2011 Jan 11;9:2. doi: 10.1186/1741-7015-9-2 (PMC3029215; doi:10.1186/1741-7015-9-2)
Supplement: Additional file 3 — Single gene differences at FDR <.05 stringency level showing AA >CA*. [file 1741-7015-9-2-S3.PDF]

Additional file 3. Single gene differences at FDR < .05 stringency level forAA>CA *

Index	Affymetrix ID	Gene Name	FDR	Fold Change (AA versus CA)	Welch t-test (P)	Description
1	205048_s_at	*PSPH*	0	4.08	$4x10^{-9}$	phosphoserine phosphatase
2	202203_s_at	*AMFR*	0	1.72	$4x10^{-6}$	autocrine motility factor receptor
3	212777_at	*SOS1*	0	1.36	$1x10^{-7}$	son of sevenless homolog 1 (Drosophila)
4	204379_s_at	*FGFR3*	0	1.24	$2X10^{-5}$	fibroblast growth factor receptor 3 (achondroplasia, thanatophoric dwarfism)
5	206777_s_at	*CRYBB2*	0.115	1.73	$5X10^{-5}$	crystallin, beta B2
6	209069_s_at	*H3F3B*	0.115	1.2	$2.2X10^{-4}$	H3 histone, family 3B (H3.3B)
7	200676_s_at	*UBE2L3*	0.115	1.19	$7X10^{-5}$	ubiquitin-conjugating enzyme E2L 3
8	201580_s_at	*DJ971N18.2*	0.115	1.18	$4X10^{-5}$	Hypothetical protein DJ971N18.2
9	200857_s_at	*NCOR1*	0.115	1.14	$1X10^{-4}$	nuclear receptor co-repressor 1
10	202695_s_at	*STK17A*	0.223	1.25	$1.7X10^{-4}$	serine/threonine kinase 17a (apoptosis-inducing)
11	218682_s_at	*SLC4A1AP*	0.223	1.23	$8X10^{-5}$	solute carrier family 4 (anion exchanger), member 1, adaptor protein
12	203532_x_at	*CUL5*	0.223	1.2	$1X10^{-4}$	cullin 5
13	213179_at	*RQCD1*	0.223	1.18	$1X10^{-4}$	RCD1 required for cell differentiation1 homolog (S. pombe) [BLAST]
14	210981_s_at	*GRK6*	0.223	1.13	$9X10^{-5}$	G protein-coupled receptor kinase 6
15	206468_s_at	*CGI-01*	0.223	1.11	$2X10^{-4}$	KIAA0859
16	206710_s_at	*EPB41L3*	0.399	2.32	$3.8X10^{-4}$	erythrocyte membrane protein band 4.1-like 3
17	218131_s_at	*p66alpha*	0.399	1.2	$2.4X10^{-4}$	P66 alpha
18	212415_at	*SEPTIN6*	0.399	1.14	$6X10^{-5}$	septin 6
19	212227_x_at	*SUI1*	0.399	1.14	$8X10^{-4}$	Putative translation initiation factor
20	212130_x_at	*SUI1*	0.399	1.12	$5.1X10^{-4}$	Putative translation initiation factor
21	207951_at	*CSN2*	0.399	1.11	$9X10^{-5}$	casein beta

*Differences in single gene expression by BOEC from AA (n=21) versus CA (n=17) subjects.
This table only shows transcripts for which AA>CA by SAM (Significance Analysis of Microarrays) and that have FDR <.05.
Genes are ranked by FDR q-value. Rank within a given FDR is by fold change. n= 21 probe sets, representing 20 genes.
