# Supplementary material for: Differential endothelial cell gene expression by African Americans versus Caucasian Americans: a possible contribution to health disparity in vascular disease and cancer
Source: BMC Med. 2011 Jan 11;9:2. doi: 10.1186/1741-7015-9-2 (PMC3029215; doi:10.1186/1741-7015-9-2)
Supplement: Additional file 4 — Single gene differences at FDR <.05 stringency level showing AA <CA*. [file 1741-7015-9-2-S4.PDF]

Additional file 4. Single gene differences at FDR < .05 for AA<CA *

Index	Affymetrix ID	Gene Name	FDR	Fold Change (AA versus CA)	Welch t-test (P)	Description
1	219417_s_at	*FLJ20014*	0	0.91	$6x10^{-5}$	Hypothetical protein FLJ20014
2	208416_s_at	*SPTB*	0	0.91	$1.9x10^{-4}$	spectrin, beta, erythrocytic (includes spherocytosis, clinical type I)
3	206763_at	*FKBP6*	0	0.9	$6x10^{-5}$	FK506 binding protein 6, 36kDa
4	202253_s_at	*DNM2*	0	0.9	$2.3x10^{-4}$	dynamin 2
5	214380_at	*PRPF31*	0	0.9	$9x10^{-5}$	PRP31 pre-mRNA processing factor 31 homolog (yeast)
6	213209_at	*TAF6L*	0	0.89	$8x10^{-7}$	TAF6-like RNA polymerase II, p300/CBP-associated factor (PCAF)-associated factor, 65kDa
7	216204_at	*ARVCF*	0	0.89	$4x10^{-6}$	armadillo repeat gene deletes in velocardiofacial syndrome
8	213585_s_at	*PDCD2*	0	0.89	$3x10^{-5}$	programmed cell death 2
9	201572_x_at	*DCTD*	0	0.89	$2.1x10^{-4}$	dCMP deaminase
10	203589_s_at	*TFDP2*	0	0.89	$3.3x10^{-4}$	transcription factor Dp2 (E2F dimerization partner 2)
11	211096_at	*PBX2*	0	0.88	$3x10^{-6}$	pre-B-cell leukemia transcription factor 2
12	215844_at	*TNPO2*	0	0.88	$8x10^{-5}$	transportin 2 (importin 3, karyopherin beta 2b) [BLAST]
13	206182_at	*ZNF134*	0	0.88	$2.3x10^{-4}$	zinc finger protein 134 (clone pHZ-15)
14	219125_s_at	*LOC55974*	0	0.87	$2x10^{-5}$	Stromal cell protein
15	201557_at	*VAMP2*	0	0.87	$6x10^{-5}$	vesicle-associated membrane protein 2 (synaptobrevin)
16	202700_s_at	*KIAA0792*	0	0.87	$1.3x10^{-4}$	KIAA0792 gene product
17	209009_at	*ESD*	0	0.87	$2x10^{-4}$	esterase D/formylglutathione hydrolase
18	200076_s_at	*MGC2749*	0	0.86	$3x10^{-5}$	Chromosome 19 open reading frame 50 FLJ25480
19	207415_at	*PLA2R1*	0	0.86	$2x10^{-5}$	phospholipase A2 receptor 1, 180kDa

20	222129_at	*C2orf17*	0	0.86	$1.6x10^{-4}$	chromosome 2 open reading frame 17
21	202646_s_at	*D1S155E*	0	0.85	$1.1x10^{-4}$	Upstream of NRAS
22	222199_s_at	*BIN3*	0	0.85	$7x10^{-5}$	bridging integrator 3
23	219939_s_at	*D1S155E*	0	0.82	$1x10^{-4}$	Upstream of NRAS
24	203538_at	*CAMLG*	0	0.82	$7x10^{-4}$	calcium modulating ligand
25	208757_at	*HSGP25L2G*	0	0.81	$1x10^{-6}$	Gp25L2 protein
26	218354_at	*LOC51693*	0	0.81	$3.5x10^{-4}$	Hematopoietic stem/progenitor cells 176
27	204340_at	*CXorf12*	0	0.77	$2x10^{-4}$	chromosome X open reading frame 12
28	219599_at	*EIF4B*	0	0.76	$4x10^{-5}$	Eukaryotic translation initiation factor 4B [BLAST]
29	219695_at	*SMPD3*	0.0115	0.92	$2.4x10^{-4}$	sphingomyelin phosphodiesterase 3, neutral membrane (neutral sphingomyelinase II) [BLAST]
30	217439_at		0.0115	0.92	$2.8x10^{-4}$	MRNA; cDNA DKFZp434L098 (from clone DKFZp434L098)
31	204895_x_at	*MUC4*	0.0115	0.92	$1.8x10^{-4}$	mucin 4, tracheobronchial [BLAST]
32	207882_at	*HSAJ2425*	0.0115	0.92	$3.3x10^{-4}$	P65 protein
33	221576_at	*GDF15*	0.0115	0.91	$5x10^{-5}$	growth differentiation factor 15 [BLAST]
34	214420_s_at	*CYP2C9*	0.0115	0.91	$3.1x10^{-4}$	cytochrome P450, family 2, subfamily C, polypeptide 9
35	202835_at	*TXNL4*	0.0115	0.91	$4.7x10^{-4}$	thioredoxin-like 4A
36	214230_at	*CDC42*	0.0115	0.9	$3x10^{-5}$	Hypothetical protein MGC16279
37	216980_s_at	*SPN*	0.0115	0.9	$1.1x10^{-4}$	sialophorin (gpL115, leukosialin, CD43)
38	210782_x_at	*GRIN1*	0.0115	0.9	$1.7x10^{-4}$	glutamate receptor, ionotropic, N-methyl D-
39	216285_at	*DGCR14*	0.0115	0.9	$2.6x10^{-4}$	DiGeorge syndrome critical region gene 14
40	64438_at	*FLJ22222*	0.0115	0.9	$1.9x10^{-4}$	Hypothetical protein FLJ22222
41	214833_at	*KIAA0792*	0.0115	0.9	$1.7x10^{-4}$	KIAA0792 gene product
42	215860_at	*SYT12*	0.0115	0.9	$2.7x10^{-4}$	synaptotagmin XII
43	205589_at	*MYL3*	0.0115	0.9	$3.3x10^{-4}$	myosin, light polypeptide 3, alkali; ventricular, skeletal, slow
44	206865_at	*HRK*	0.0115	0.9	$3.1x10^{-4}$	harakiri, BCL2 interacting protein (contains only BH3 domain)

45	56919_at	*GORASP1*	0.0115	0.89	4.9×10^{-4}	WD repeat endosomal protein
46	40829_at	*WDTC1*	0.0115	0.89	2.6×10^{-4}	WD and tetratricopeptide repeats 1
47	220096_at	*RNASET2*	0.0115	0.89	2.3×10^{-4}	ribonuclease T2
48	202452_at	*C9orf60*	0.0115	0.89	7.2×10^{-4}	chromosome 9 open reading frame 60
49	203421_at	*TP53I11*	0.0115	0.89	3.7×10^{-4}	tumor protein p53 inducible protein 11
50	218328_at	*COQ4*	0.0115	0.89	9.5×10^{-4}	coenzyme Q4 homolog (yeast)
51	203488_at	*LPHN1*	0.0115	0.89	2.8×10^{-4}	latrophilin 1
52	222123_s_at	*HIF3A*	0.0115	0.89	2.9×10^{-4}	hypoxia inducible factor 3, alpha subunit
53	202117_at	*ARHGAP1*	0.0115	0.89	8.6×10^{-4}	Rho GTPase activating protein 1
54	207321_s_at	*ABCB9*	0.0115	0.89	3.2×10^{-4}	ATP-binding cassette, sub-family B (MDR/TAP),
55	211230_s_at	*PIK3CD*	0.0115	0.89	3.2×10^{-4}	Phosphoinositide-3-kinase, catalytic, delta
56	203759_at	*SIAT4C*	0.0115	0.88	3.3×10^{-4}	sialyltransferase 4C (beta-galactoside alpha-2,3-sialyltransferase)
57	213350_at	*RPS11*	0.0115	0.88	3.4×10^{-4}	ribosomal protein S11
58	202028_s_at	*RPL38*	0.0115	0.88	7×10^{-4}	ribosomal protein L38
59	215667_x_at	*PMS2L5*	0.0115	0.88	1.14×10^{-3}	Postmeiotic segregation increased 2-like 2
60	203926_x_at	*ATP5D*	0.0115	0.87	8.1×10^{-4}	ATP synthase, H+ transporting, mitochondrial F1 complex, delta subunit
61	217254_s_at	*EPO*	0.0115	0.87	1.9×10^{-4}	erythropoietin
62	208946_s_at	*BECN1*	0.0115	0.87	6.9×10^{-4}	beclin 1 (coiled-coil, myosin-like BCL2 interacting protein)
63	208238_x_at		0.0115	0.86	3.4×10^{-4}	no annotation available
64	221071_at		0.0115	0.85	4.5×10^{-4}	no annotation available
65	201949_x_at	*CAPZB*	0.0115	0.85	5.7×10^{-4}	capping protein (actin filament) muscle Z-line, beta
66	218531_at	*FLJ21749*	0.0115	0.85	8×10^{-4}	Hypothetical protein FLJ21749
67	210059_s_at	*MAPK13*	0.0115	0.85	8×10^{-4}	mitogen-activated protein kinase 13
68	217812_at	*YTHDF2*	0.0115	0.84	6.6×10^{-4}	YTH domain family, member 2
69	221834_at	*LONP*	0.0115	0.82	4.5×10^{-4}	seven in absentia homolog 1 (Drosophila)
70	221892_at	*H6PD*	0.0115	0.82	4.6×10^{-4}	hexose-6-phosphate dehydrogenase (glucose
71	206332_s_at	*IFI16*	0.0115	0.82	1.41×10^{-3}	interferon, gamma-inducible protein 16

72	220753_s_at	*CRYL1*	0.0115	0.72	1.65×10^{-3}	crystallin, lambda 1
73	221287_at	*RNASEL*	0.0223	0.93	3.8×10^{-4}	ribonuclease L (2',5'-oligoisoadenylate synthetase-dependent)
74	205623_at	*ALDH3A1*	0.0223	0.93	4.1×10^{-4}	aldehyde dehydrogenase 3 family, memberA1
75	215376_at		0.0223	0.93	3.4×10^{-4}	CDNA FLJ12295 fis, clone MAMMA1001818
76	214284_s_at	*FGF18*	0.0223	0.92	4.2×10^{-4}	fibroblast growth factor 18
77	213749_at	*MASP1*	0.0223	0.92	5.5×10^{-4}	mannan-binding lectin serine protease 1 (C4/C2 activating component of Ra-reactive factor)
78	207634_at	*PDCD1*	0.0223	0.92	1.29×10^{-3}	programmed cell death 1
79	208212_s_at	*ALK*	0.0223	0.91	5.8×10^{-4}	anaplastic lymphoma kinase (Ki-1)
80	207118_s_at	*MMP23B*	0.0223	0.91	5.2×10^{-4}	Matrix metalloproteinase 23B
81	210107_at	*CLCA1*	0.0223	0.91	9×10^{-4}	chloride channel,calcium activated, family member1
82	39817_s_at	*C6orf108*	0.0223	0.91	9.4×10^{-4}	chromosome 6 open reading frame 108
83	209881_s_at	*LAT*	0.0223	0.91	7.3×10^{-4}	linker for activation of T cells
84	220570_at	*RETN*	0.0223	0.91	5×10^{-4}	resistin
85	216495_x_at		0.0223	0.91	8.8×10^{-4}	IgG to Puumala virus G2, light chain variable region
86	220425_x_at	*ROPN1*	0.0223	0.9	5.4×10^{-4}	Similar to AKAP-binding sperm protein ropporin [BLAST]
87	214125_s_at	*SPUF*	0.0223	0.9	4.2×10^{-4}	Secreted protein of unknown function
88	221206_at	*PMS2*	0.0223	0.9	1.24×10^{-3}	Similar to PMS1 protein homolog 2 (DNA mismatch repair protein PMS2) [BLAST]
89	213395_at	*MLC1*	0.0223	0.9	5.3×10^{-4}	megalencephalic leukoencephalopathy with subcortical cysts 1 [BLAST]
90	213202_at	*KIAA0339*	0.0223	0.9	1.07×10^{-3}	KIAA0339 gene product
91	210314_x_at	*TNFSF13*	0.0223	0.9	8.5×10^{-4}	Tumor necrosis factor (ligand) superfamily, member 12
92	214000_s_at	*RGS10*	0.0223	0.9	1.14×10^{-3}	regulator of G-protein signalling 10
93	220709_at	*ZNF556*	0.0223	0.9	7.4×10^{-4}	zinc finger protein 556
94	208041_at	*GRK1*	0.0223	0.89	8.7×10^{-4}	G protein-coupled receptor kinase 1
95	214171_s_at	*U2AF2*	0.0223	0.89	5.9×10^{-4}	U2 (RNU2) small nuclear RNA auxiliary factor 2

96	206217_at	*ED1*	0.0223	0.89	5.4×10^{-4}	ectodysplasin A
97	221592_at	*TBC1D8*	0.0223	0.89	9.4×10^{-4}	TBC1 domain family, member8 (with GRAM domain)
98	216331_at	*ITGA7*	0.0223	0.89	5.3×10^{-4}	integrin, alpha 7
99	215273_s_at	*TADA3L*	0.0223	0.89	1.44×10^{-3}	transcriptional adaptor 3 (NGG1 homolog, yeast)-like
100	204636_at	*COL17A1*	0.0223	0.89	1.11×10^{-3}	collagen, type XVII, alpha 1
101	214918_at	*HNRPM*	0.0223	0.89	8.2×10^{-4}	heterogeneous nuclear ribonucleoprotein M
102	219441_s_at	*LRRK1*	0.0223	0.88	5.4×10^{-4}	leucine-rich repeat kinase 1
103	206646_at	*GLI*	0.0223	0.88	4.1×10^{-4}	glioma-associated oncogene homolog (zinc finger dependent)
104	200026_at	*RPL34*	0.0223	0.88	2.04×10^{-3}	Ribosomal protein L34
105	220977_x_at	*EPB41L5*	0.0223	0.88	1.34×10^{-3}	erythrocyte membrane protein band 4.1 like 5
106	210483_at	*MGC31957*	0.0223	0.87	4.1×10^{-4}	Tumor necrosis factor receptor superfamily, member 10c, decoy without an intracellular domain
107	206824_at	*CES1*	0.0223	0.87	5×10^{-4}	carboxylesterase 4-like
108	213963_s_at	*SAP30*	0.0223	0.87	9.3×10^{-4}	sin3-associated polypeptide, 30kDa
109	217852_s_at	*ARL10C*	0.0223	0.86	1.1×10^{-3}	ADP-ribosylation factor-like 10C
110	220741_s_at	*PPA2*	0.0223	0.85	8.2×10^{-4}	Inorganic pyrophosphatase 2
111	217945_at	*BTBD1*	0.0223	0.85	1.65×10^{-3}	BTB (POZ) domain containing 1
112	216609_at	*TXN*	0.0223	0.84	7.3×10^{-4}	thioredoxin
113	215600_x_at	*LOC285231*	0.0223	0.84	1×10^{-3}	F-box and WD-40 domain protein 12
114	218821_at	*NPEPL1*	0.0223	0.83	2.77×10^{-3}	aminopeptidase-like 1 [BLAST]
115	218034_at	*TTC11*	0.0223	0.82	2.08×10^{-3}	tetratricopeptide repeat domain 11
116	219660_s_at	*ATP8A2*	0.0223	0.76	5.52×10^{-3}	ATPase, aminophospholipid transporter-like, Class I,
117	217122_s_at	*SLC35E2*	0.0223	0.77	1.98×10^{-3}	Hypothetical gene supported by AK097814
118	201810_s_at	*SH3BP5*	0.0223	0.74	3.52×10^{-3}	SH3-domain binding protein 5 (BTK-associated)
119	207416_s_at	*NFATC3*	0.039	0.94	1.66×10^{-3}	nuclear factor of activated T-cells, cytoplasmic, calcineurin-dependent 3
120	217311_at		0.039	0.93	1.09×10^{-3}	Homo sapiens aconitase precursor (ACON) mRNA, nuclear gene encoding mitochondrial protein, partial cds

121	215403_at		0.039	0.93	$1.47x10^{-3}$	triple functional domain (PTPRF interacting)
122	221941_at	*PAOX*	0.039	0.93	$2.73x10^{-3}$	polyamine oxidase (exo-N4-amino)
123	217066_s_at	*DMPK*	0.039	0.93	$2.4x10^{-3}$	dystrophia myotonica-protein kinase
124	203863_at	*ACTN2*	0.039	0.93	$6.8x10^{-4}$	actinin, alpha 2
125	212939_at	*COL6A1*	0.039	0.93	$2.56x10^{-3}$	collagen, type VI, alpha 1
126	206146_s_at	*RHAG*	0.039	0.92	$1.51x10^{-3}$	Rhesus blood group-associated glycoprotein
127	215544_s_at	*UBCE7IP5*	0.039	0.92	$1.19x10^{-3}$	Likely ortholog of mouse ubiquitin conjugating enzyme 7 interacting protein 5 [BLAST]
128	212700_x_at	*PLEKHM1*	0.039	0.92	$1.51x10^{-3}$	pleckstrin homology domain containing, family M (with RUN domain) member 1
129	221812_at	*FBXO42*	0.039	0.92	$2.02x10^{-3}$	F-box protein 42
130	220675_s_at	*C22orf20*	0.039	0.92	$9x10^{-4}$	adiponutrin
131	214798_at	*KIAA0703*	0.039	0.92	$1.01x10^{-3}$	KIAA0703 gene product
132	212790_x_at	*RPL13A*	0.039	0.92	$2.59x10^{-3}$	ribosomal protein L13a
133	215693_x_at	*DDX27*	0.039	0.92	$2.03x10^{-3}$	DEAD (Asp-Glu-Ala-Asp) box polypeptide 27
134	200688_at	*SF3B3*	0.039	0.92	$1.9x10^{-3}$	splicing factor 3b, subunit 3, 130kDa
135	217270_s_at	*DYRK1B*	0.039	0.92	$1.88x10^{-3}$	dual-specificity tyrosine-(Y)-phosphorylation regulated kinase 1B
136	34846_at	*CAMK2B*	0.039	0.92	$1.67x10^{-3}$	calcium/calmodulin-dependent protein kinase (CaM kinase) II beta
137	207699_at	*ZNF409*	0.039	0.92	$1.63x10^{-3}$	zinc finger protein 409
138	221187_s_at	*FLJ22688*	0.039	0.92	$1.45x10^{-3}$	Hypothetical protein FLJ22688
139	216727_at	*STK38*	0.039	0.92	$8.5x10^{-4}$	serine/threonine kinase 38
140	213818_x_at	*LOC374395*	0.039	0.92	$2.22x10^{-3}$	Similar to RIKEN cDNA 1810059G22
141	205853_at	*ZFP67*	0.039	0.92	$1.61x10^{-3}$	zinc finger protein 67 homolog (mouse)
142	204870_s_at	*PCSK2*	0.039	0.91	$5x10^{-4}$	proprotein convertase subtilisin/kexin type 2
143	221259_s_at	*TEX11*	0.039	0.91	$5.5x10^{-4}$	testis expressed sequence 11
144	210128_s_at	*LTB4R*	0.039	0.91	$9.8x10^{-4}$	leukotriene B4 receptor
145	219319_at	*HIF3A*	0.039	0.91	$1.82x10^{-3}$	hypoxia inducible factor 3, alpha subunit
146	206333_at	*MSI1*	0.039	0.91	$8.2x10^{-4}$	musashi homolog 1 (Drosophila)

147	202876_s_at	*PBX2*	0.039	0.91	$1.46x10^{-3}$	pre-B-cell leukemia transcription factor 2
148	81737_at		0.039	0.91	$2.31x10^{-3}$	Similar to Group X secretory phospholipase A2 precursor (Phosphatidylcholine 2-acylhydrolase GX) (GX sPLA2) (sPLA2-X)
149	212929_s_at	*FLJ10824*	0.039	0.91	$3.21x10^{-3}$	Similar to KIAA0592 protein
150	206436_at	*C22orf1*	0.039	0.91	$2x10^{-3}$	chromosome 22 open reading frame 1
151	214471_x_at	*CGB*	0.039	0.9	$9x10^{-4}$	luteinizing hormone beta polypeptide [BLAST]
152	220488_s_at	*BCAS3*	0.039	0.9	$1.57x10^{-3}$	breast carcinoma amplified sequence 3
153	215553_x_at		0.039	0.9	$1.22x10^{-3}$	WD repeat domain 45
154	216835_s_at	*DOK1*	0.039	0.9	$1.87x10^{-3}$	docking protein 1, 62kDa (downstream of tyrosine
155	220371_s_at	*TRIP6*	0.039	0.9	$1.68x10^{-3}$	solute carrier family 12 (potassium/chloride transporters), member 9 [BLAST]
156	216611_s_at	*SLC6A2*	0.039	0.9	$1.16x10^{-3}$	solute carrier family 6 (neurotransmitter transporter, noradrenalin), member 2
157	222293_at	*IGSF4C*	0.039	0.9	$1.11x10^{-3}$	immunoglobulin superfamily, member 4C
158	220519_s_at	*LIM2*	0.039	0.9	$1.28x10^{-3}$	lens intrinsic membrane protein 2, 19kDa
159	209918_at	*GGT1*	0.039	0.9	$1.08x10^{-3}$	gamma-glutamyltransferase 1
160	208299_at	*CACNA1I*	0.039	0.9	$1.3x10^{-3}$	calcium channel, voltage-dependent,alpha 1I subunit
161	208232_x_at	*NRG1*	0.039	0.9	$1.47x10^{-3}$	neuregulin 1
162	217040_x_at	*SOX15*	0.039	0.9	$1.82x10^{-3}$	SRY (sex determining region Y)-box 15
163	208902_s_at	*FLJ46061*	0.039	0.9	$2.77x10^{-3}$	FLJ46061 protein
164	218909_at	*RPS6KC1*	0.039	0.89	$2.46x10^{-3}$	ribosomal protein S6 kinase, 52kDa, polypeptide 1
165	209438_at	*PHKA2*	0.039	0.89	$1.03x10^{-3}$	phosphorylase kinase, alpha 2 (liver)
166	200932_s_at	*DCTN2*	0.039	0.89	$3.19x10^{-3}$	dynactin 2 (p50)
167	204660_at	*GFER*	0.039	0.89	$1.06x10^{-3}$	growth factor, augmenter of liver regeneration
168	206093_x_at	*TNXB*	0.039	0.89	$1.16x10^{-3}$	tenascin XB [BLAST]
169	220691_at	*PRO0097*	0.039	0.89	$1.03x10^{-3}$	transmembrane protein 23 [BLAST]
170	206328_at	*CDH15*	0.039	0.89	$1.03x10^{-3}$	cadherin 15, M-cadherin (myotubule)
171	208034_s_at	*PROZ*	0.039	0.89	$3.16x10^{-3}$	protein Z, vitamin K-dependent plasma glycoprotein
172	209425_at	*AMACR*	0.039	0.89	$1.11x10^{-3}$	alpha-methylacyl-CoA racemase

173	214122_at	*PDLIM7*	0.039	0.89	$1.59x10^{-3}$	PDZ and LIM domain 7 (enigma)
174	208634_s_at	*MACF1*	0.039	0.89	$2.41x10^{-3}$	Microtubule-actin crosslinking factor 1
175	205056_s_at	*GRCA*	0.039	0.89	$1.39x10^{-3}$	Gene rich cluster, A gene
176	204292_x_at	*STK11*	0.039	0.89	$1.83x10^{-3}$	serine/threonine kinase 11 (Peutz-Jeghers syndrome)
177	214223_at	*PTP4A3*	0.039	0.89	$1.96x10^{-3}$	protein tyrosine phosphatase type IVA, member 3
178	217765_at	*NRBP*	0.039	0.88	$2.41x10^{-3}$	nuclear receptor binding protein [BLAST]
179	216859_x_at		0.039	0.88	$1.17x10^{-3}$	Homo sapiens genomic DNA; cDNA DKFZp586H0722 (from clone DKFZp586H0722)
180	219379_x_at	*ZNF358*	0.039	0.88	$3.83x10^{-3}$	zinc finger protein 358
181	221419_s_at		0.039	0.88	$2.49x10^{-3}$	Similar to CG7467-PA
182	221013_s_at	*APOL2*	0.039	0.88	$1.98x10^{-3}$	apolipoprotein L, 2
183	212567_s_at	*MAP4*	0.039	0.88	$3.56x10^{-3}$	microtubule-associated protein 4
184	222264_at	*DKFZp762N1*	0.039	0.87	$9.5x10^{-4}$	Hypothetical protein DKFZp762N1910
185	201644_at	*TSTA3*	0.039	0.87	$2.25x10^{-3}$	tissue specific transplantation antigen P35B
186	202821_s_at	*LPP*	0.039	0.87	$2.66x10^{-3}$	LIM domain containing preferred translocation partner in lipoma
187	219870_at	*ATF7IP2*	0.039	0.87	$4.82x10^{-3}$	activating transcription factor 7 interacting protein 2
188	209502_s_at	*BAIAP2*	0.04	0.87	$4.14x10^{-3}$	BAI1-associated protein 2
189	203683_s_at	*VEGFB*	0.039	0.86	$4.49x10^{-3}$	vascular endothelial growth factor B
190	213892_s_at	*APRT*	0.039	0.85	$1.92x10^{-3}$	adenine phosphoribosyltransferase
191	218764_at	*PRKCH*	0.039	0.85	$2.98x10^{-3}$	protein kinase C, eta [BLAST]
192	201527_at	*ATP6V1F*	0.039	0.84	$3.52x10^{-3}$	ATPase, H+ transporting, lysosomal 14kDa, V1
193	36936_at	*TSTA3*	0.039	0.84	$3.26x10^{-3}$	tissue specific transplantation antigen P35B
194	219113_x_at	*DHRS10*	0.039	0.84	$2.29x10^{-3}$	dehydrogenase/reductase (SDR family) member 10
195	213398_s_at	*C14orf124*	0.039	0.83	$4.51x10^{-3}$	chromosome 14 open reading frame 124
196	222138_s_at	*WDR13*	0.039	0.83	$6.97x10^{-3}$	WD repeat domain 13 [BLAST]
197	202271_at	*FBXO28*	0.039	0.81	$4.81x10^{-3}$	F-box protein 28
198	210778_s_at	*MXD4*	0.039	0.8	$4.3x10^{-3}$	MAX dimerization protein 4
199	37966_at	*PARVB*	0.039	0.79	$1.61x10^{-3}$	parvin, beta

200	217761_at	*SIPL*	0.039	0.76	1.67×10^{-3}	Membrane-type 1 matrix metalloproteinase cytoplasmic tail binding protein-1

*Differences in single gene expression by BOEC from AA (n=21) versus CA (n=17) subjects.
This table only shows transcripts for which AA<CA by SAM (Significance Analysis of Microarrays) and that have FDR <.05.
Genes are ranked by FDR q-value. Rank within a given FDR is by fold change. n= 200 probe sets, representing 184 genes.
