# Supplementary material for: Differential endothelial cell gene expression by African Americans versus Caucasian Americans: a possible contribution to health disparity in vascular disease and cancer
Source: BMC Med. 2011 Jan 11;9:2. doi: 10.1186/1741-7015-9-2 (PMC3029215; doi:10.1186/1741-7015-9-2)
Supplement: Additional file 5 — Shear stress biology gene set - expanded gene names. [file 1741-7015-9-2-S5.PDF]

Additional File 5. Shear stress biology gene set -- expanded gene names

Part A. Gene Contributes to Core Enrichment. "Core enrichment" indicates that the corresponding gene is in the leading edge subset, ie, the subset of genes that contributes most significantly to that biological system's results using GSEA (Table 2) in the text. In the table below, entries are listed in alphabetical order.

**Gene Name**	**Gene Description**
BMP4	bone morphogenetic protein 4
BMP6	bone morphogenetic protein 6
CCL2	chemokine (C-C motif) ligand 2
CD34	CD34 antigen
CENPF	centromere protein F, 350/400ka (mitosin)
CTGF	connective tissue growth factor
CXCL12	chemokine (C-X-C motif) ligand 12 (stromal cell-derived factor 1)
CYP1B1	cytochrome P450, family 1, subfamily B, polypeptide 1
CYR61	cysteine-rich, angiogenic inducer, 61
DPYSL3	dihydropyrimidinase-like 3
EDN1	endothelin 1
EEF1A2	eukaryotic translation elongation factor 1 alpha 2
ELN	elastin (supravalvular aortic stenosis, Williams-Beuren syndrome)
F3	coagulation factor III (thromboplastin, tissue factor)
FGF2	fibroblast growth factor 2 (basic)
FN1	fibronectin 1 [BLAST]
GAPD	glyceraldehyde-3-phosphate dehydrogenase
GBP1	guanylate binding protein 1, interferon-inducible, 67kDa
GJA5	gap junction protein, alpha 5, 40kDa (connexin 40)
GSTP1	glutathione S-transferase pi
IL8	interleukin 8
ILK	Integrin-linked kinase
JAG2	jagged 2
JUNB	jun B proto-oncogene
KIF20A	kinesin family member 20A
KLF4	Kruppel-like factor 4 (gut)
LIG3	ligase III, DNA, ATP-dependent
MAPRE1	microtubule-associated protein, RP/EB family, member 1
MATN2	matrilin 2
MYC	v-myc myelocytomatosis viral oncogene homolog (avian)
NOS3	nitric oxide synthase 3 (endothelial cell)
PFDN2	prefoldin 2
PLAT	plasminogen activator, tissue
RGS3	regulator of G-protein signalling 3
RHOA	ras homolog gene family, member A
RHOB	ras homolog gene family, member B
S100A10	S100 calcium binding protein A10 (annexin II ligand, calpactin I, light polypeptide (p11)
SP1	Sp1 transcription factor

TBXAS1	thromboxane A synthase 1 (platelet, cytochrome P450, family 5, subfamily A)
TGFB1	transforming growth factor, beta 1 (Camurati-Engelmann disease)
THBD	thrombomodulin
THBS1	thrombospondin 1
TRA1	tumor rejection antigen (gp96) 1
TUBG1	tubulin, gamma 1
VCAM1	vascular cell adhesion molecule 1
VIPR1	vasoactive intestinal peptide receptor 1
XPO1	exportin 1 (CRM1 homolog, yeast)

Part B. Gene Does Not Contribute to Enrichment Score

**Gene Name**	**Gene Description**
ACTB	actin, beta
AKAP1	A kinase (PRKA) anchor protein 1
ANXA2	annexin A2
ANXA5	annexin A5
APOE	apolipoprotein E
APS	Adaptor protein with pleckstrin homology and src homology 2 domains
ARF4L	ADP-ribosylation factor 4-like
ASS	argininosuccinate synthetase
CAV1	caveolin 1, caveolae protein, 22kDa
CCL15	chemokine (C-C motif) ligand 14 [BLAST]
CCL25	chemokine (C-C motif) ligand 25
CD164	CD164 antigen, sialomucin
CD1D	CD1D antigen, d polypeptide
CD58	CD58 antigen, (lymphocyte function-associated antigen 3)
CD68	CD68 antigen
CDKN1A	cyclin-dependent kinase inhibitor 1A (p21, Cip1)
CEACAM1	carcinoembryonic antigen-related cell adhesion molecule 1 (biliary glycoprotein)
CYP1A1	cytochrome P450, family 1, subfamily A, polypeptide 1
DKK2	dickkopf homolog 2 (Xenopus laevis)
E2F5	E2F transcription factor 5, p130-binding
EDN3	endothelin 3
EFEMP1	EGF-containing fibulin-like extracellular matrix protein 1
EGR1	early growth response 1
EIF4EBP2	eukaryotic translation initiation factor 4E binding protein 2 [BLAST]
EIF4G3	eukaryotic translation initiation factor 4 gamma, 3
ESM1	endothelial cell-specific molecule 1
F2	coagulation factor II (thrombin)
F2R	coagulation factor II (thrombin) receptor
FGF6	fibroblast growth factor 6
FGFR3	fibroblast growth factor receptor 3 (achondroplasia, thanatophoric dwarfism)
FLT1	fms-related tyrosine kinase 1 (vascular endothelial growth factor/vascular permeability factor receptor)
FOS	v-fos FBJ murine osteosarcoma viral oncogene homolog

FOSL1	FOS-like antigen 1
FTH1	ferritin, heavy polypeptide 1
FTL	ferritin, light polypeptide
GARS	glycyl-tRNA synthetase
GAS	gastrin
GJA1	gap junction protein, alpha 1, 43kDa (connexin 43)
GNAS	GNAS complex locus
GNB2	guanine nucleotide binding protein (G protein), beta polypeptide 2
GNG5	guanine nucleotide binding protein (G protein), gamma 5
GRN	granulin
HADHSC	adducin 3 (gamma)
HMOX1	heme oxygenase (decycling) 1
ICAM1	intercellular adhesion molecule 4, Landsteiner-Wiener blood group
IFITM3	interferon induced transmembrane protein 2 (1-8D)
IL13RA1	interleukin 13 receptor, alpha 1
IL15	interleukin 15
IL16	interleukin 16 (lymphocyte chemoattractant factor)
IL1R1	interleukin 1 receptor, type I
IL1RL1	interleukin 1 receptor-like 1
ITGB3	integrin, beta 3 (platelet glycoprotein IIIa, antigen CD61)
JARID1A	Jumonji, AT rich interactive domain 1A (RBBP2-like)
JUN	v-jun sarcoma virus 17 oncogene homolog (avian)
KLF2	Kruppel-like factor 2 (lung)
LAMB1	laminin, beta 1
LIMS1	LIM and senescent cell antigen-like domains 1
LRP2	low density lipoprotein-related protein 2
METAP2	mRNA; cDNA DKFZp686F18245 (from clone DKFZp686F18245) [BLAST]
MGP	matrix Gla protein
MMP1	matrix metalloproteinase 1 (interstitial collagenase)
MMP14	matrix metalloproteinase 14 (membrane-inserted)
MMRN1	multimerin 1
MYD88	myeloid differentiation primary response gene (88)
NFKB1	nuclear factor of kappa light polypeptide gene enhancer in B-cells 1 (p105)
NOTCH4	Notch homolog 4 (Drosophila)
NQO1	NAD(P)H dehydrogenase, quinone 1
NUMA1	nuclear mitotic apparatus protein 1
OGT	O-linked N-acetylglucosamine (GlcNAc) transferase (UDP-N-acetylglucosamine:polypeptide-N-acetylglucosaminyl transferase)
OLR1	oxidised low density lipoprotein (lectin-like) receptor 1
PBP	prostatic binding protein
PCQAP	PC2 (positive cofactor 2, multiprotein complex) glutamine/Q-rich-associated protein [BLAST]
PDGFRA	platelet-derived growth factor receptor, alpha polypeptide
PDGFRB	platelet-derived growth factor receptor, beta polypeptide
PDPK1	3-phosphoinositide dependent protein kinase-1
PECAM1	platelet/endothelial cell adhesion molecule (CD31 antigen)
PFDN5	prefoldin 5

PTGS2	prostaglandin-endoperoxide synthase 2 (prostaglandin G/H synthase and cyclooxygenase)
RGS5	regulator of G-protein signalling 5
RPL30	ribosomal protein, L30
RPL34	ribosomal protein, L34
RPS11	ribosomal protein, S11
RPS7	ribosomal protein, S7
SAT	spermidine/spermine N1-acetyltransferase
SCGF	stem cell growth factor; lymphocyte secreted C-type lectin
SELE	selectin E (endothelial adhesion molecule 1)
SERPINE1	serine (or cysteine) proteinase inhibitor, clade E (nexin, plasminogen activator inhibitor type 1), member 1
SERPINE2	serine (or cysteine) proteinase inhibitor, clade E (nexin, plasminogen activator inhibitor type 1), member 2
SLC35F2	ribosomal protein, large P2
SMARCD1	SWI/SNF related, matrix associated, actin dependent regulator of chromatin, subfamily d, member 1
SOD2	superoxide dismutase 2, mitochondrial
SPARC	secreted protein, acidic, cysteine-rich (osteonectin)
SPTA1	spectrin, alpha, erythrocytic 1 (elliptocytosis 2)
SPTAN1	spectrin, alpha, non-erythrocytic 1 (alpha-fodrin)
STAM	signal transducing adaptor molecule (SH3 domain and ITAM motif) 1
TEK	TEK tyrosine kinase, endothelial (venous malformations, multiple cutaneous and mucosal)
TFPI	tissue factor pathway inhibitor (lipoprotein-associated coagulation inhibitor)
TGFB1I4	transforming growth factor beta 1 induced transcript 4
THBS4	thrombospondin 4
TNFRSF1A	tumor necrosis factor receptor superfamily, member 1A
TNFRSF5	tumor necrosis factor receptor superfamily, member 5
TNFSF8	tumor necrosis factor (ligand) superfamily, member 8
TUBA3	tubulin, alpha 3
TXNRD1	thioredoxin reductase 1
UNG	uracil-DNA glycosylase
VCL	vinculin
VEGFC	vascular endothelial growth factor C
VWF	von Willebrand factor
WNT2B	wingless-type MMTV integration site family, member 2B
